# Supplementary material for: Retargeted adenoviruses for local IgA and CD47 blocker production as a novel cancer therapy
Source: EMBO Mol Med. 2026 Jul 7;18(8):3226–57. doi: 10.1038/s44321-026-00477-7 (PMC13469128; doi:10.1038/s44321-026-00477-7)
Supplement: Supplementary file 1 [file 44321_2026_477_MOESM11_ESM.pdf]

Expanded View Figures

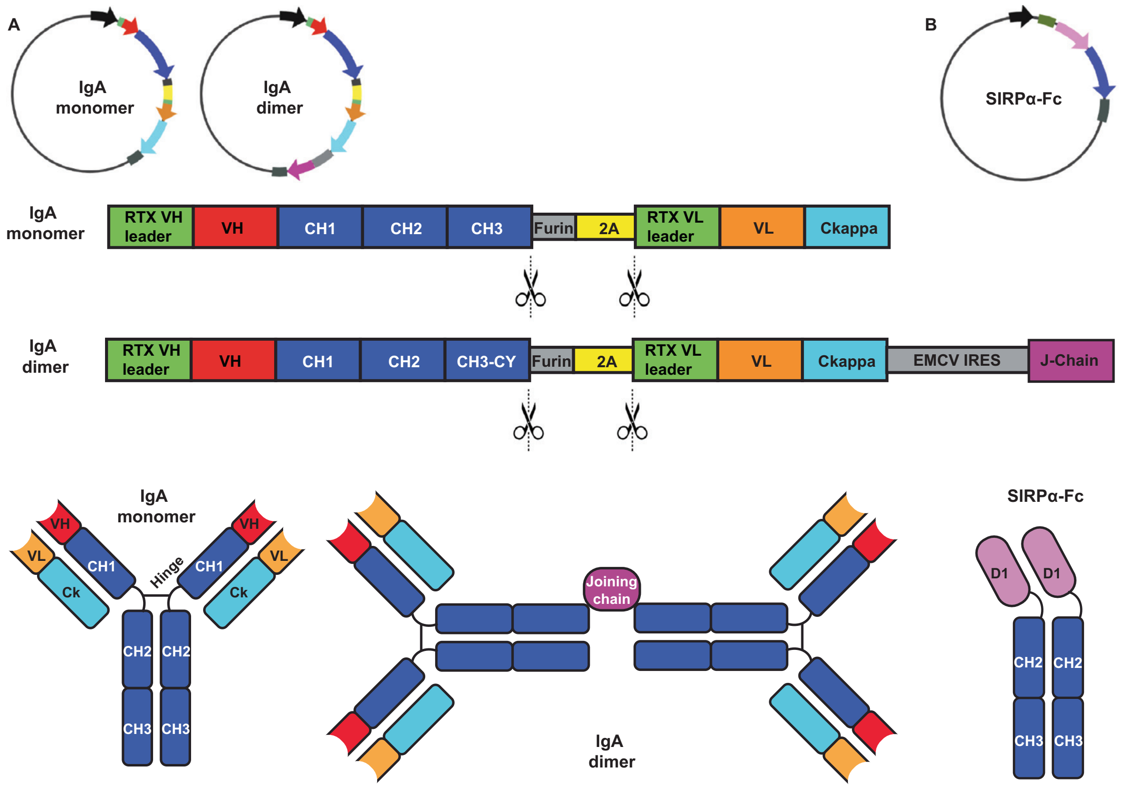

Figure EV1. Schematic representation of the engineered pShuttle expression vectors for expression of IgA and SIRPα-Fc.

(**A**) Schematic representation of the encoded IgA molecules and the corresponding pShuttle expression vector scheme maps are shown. Black: promoter (CMV); green: leader sequence from rituximab; red, orange: VH and VL variable domains of the heavy and light chain of IgA, respectively; gray: furin recognition sequence, followed by a P2A or T2A "self-cleaving" (ribosome skipping) sequence (yellow); the cleavage site is indicated schematically; blue and cyan: heavy chain and light chain of IgA with the respective CH1-CH3 heavy and VL light chain domains. Dimeric IgA was encoded in the same vector format with a modified CH3 domain (introduced C-terminal Cys-Tyr (CY) residues), with the joining (J-) chain (magenta) encoded under EMCV IRES sequence (light gray). (**B**) Schematic representation of the SIRPα-Fc molecule and the respective expression vector map. Black: EF-1α promoter; green: SIRPα leader sequence; pink: Consensus variant (CV) of D1 domain of SIRPα engineered with increased affinity to human CD47 and cross-reactivity with murine CD47; blue: Fc-ablated, AAA-mutant variant of the IgG1 heavy chain CH2-CH3 domains. RTX rituximab.

 © The Author(s)

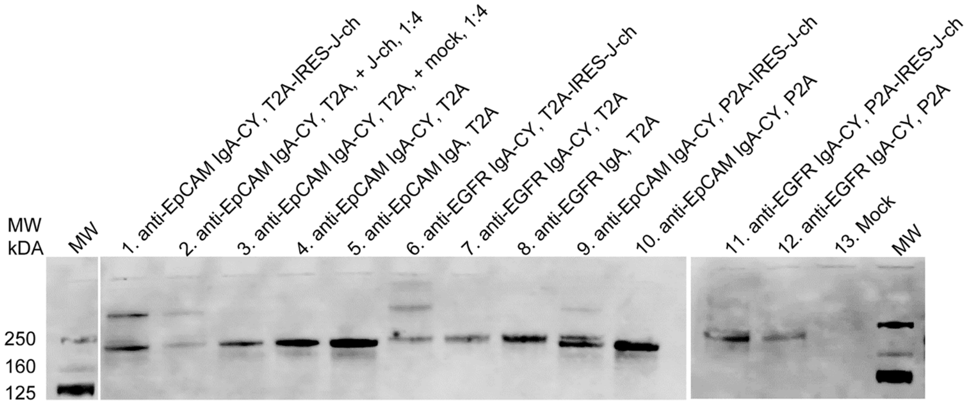

Figure EV2. Western blot analysis of dimeric IgA expressed from various expression vectors.

Supernatants of CHO-S cells transfected with the anti-EGFR or anti-EpCAM IgA expression constructs containing T2A or P2A "self-cleaving" (ribosome skipping) sequences were compared in western blotting under non-reducing conditions, by detecting IgA heavy chain (HC). Bicistronic constructs encoding monomeric IgA (T2A variants) were used as controls yielding a band corresponding to a monomeric IgA (lanes 5 and 8); the same constructs containing C-terminal Cys-Tyr (CY) residues, which are reintroduced as they are required for dimer formation, were used as controls to validate successful antibody production (lanes 4 and 7 for T2A variants, lanes 10 and 12 for P2A variants). Tricistronic vectors where the J-chain was encoded under an IRES resulted in expression of the dimeric IgA fraction detected at the height of ~350 kDa (lanes 1, 6 for T2A-variants and lanes 9, 11 for P2A variants), with the T2A variants yielding a higher proportion of the dimer compared to the monomer fraction (~ 350 kDa band vs ~170 kDa band). To investigate if supplying J-chain in a higher molar ratio (1:4 IgA construct: J-chain construct) would result in a higher dimer proportion, CHO-S cells were co-transfected with the vector encoding the IgA monomer with the reintroduced CY sequence, together with the vector encoding only the J-chain (lane 2), which resulted in a similar ratio of dimer:monomer as for the tricistronic vector (lane 1) but overall lower yield. To normalize for differences due to co-transfection, we included the control where CHO-S were co-transfected with the same IgA construct as in lane 2, but with a mock (GFP) construct instead of the J-chain, in the same molar ratios (lane 3). Finally, a mock-transfected sample (GFP) was included as a negative control (lane 13). The image is representative of three biological replicates.

© The Author(s)

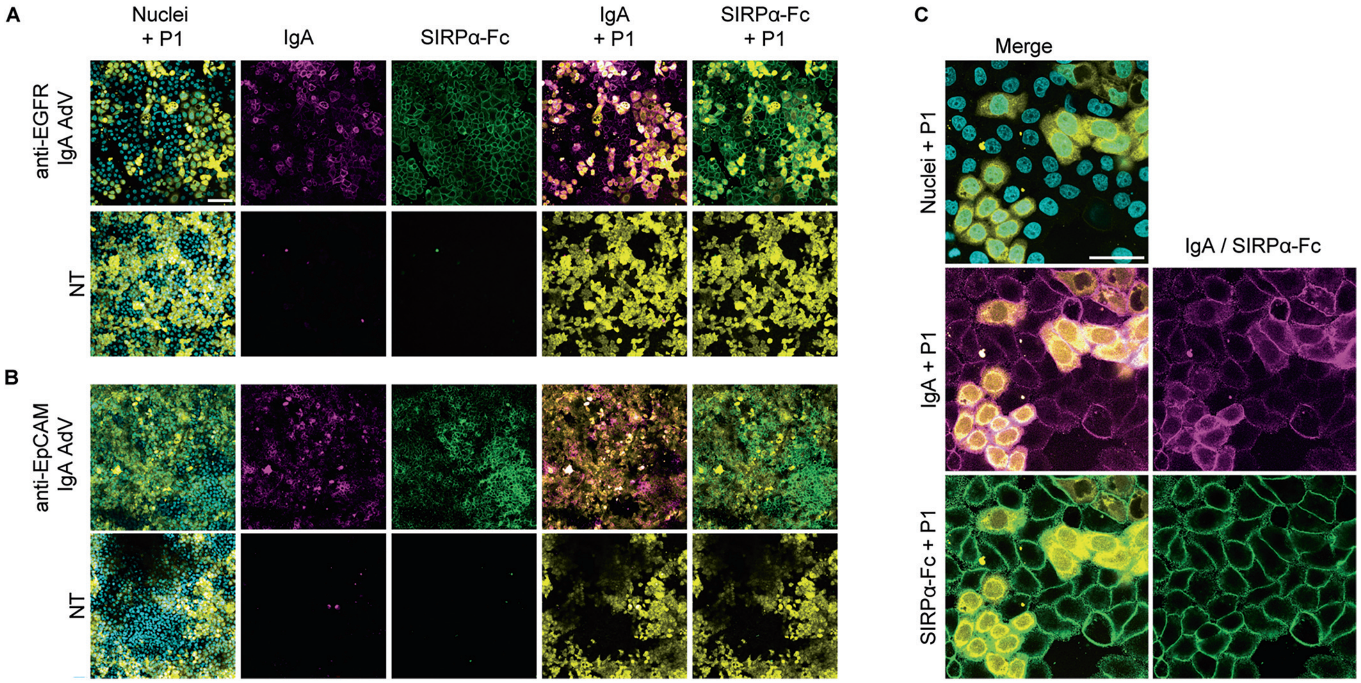

Figure EV3. IgA antibody and SIRPα-Fc genes are co-delivered with retargeted adenoviral vectors, and produced proteins bind in an autocrine and paracrine fashion to tumor cells.

SIRPα-Fc and Anti-EGFR IgA and anti-EpCAM IgA were produced in MDA-MB-468 (**A**, **C**) and HCT116 cells (**B**) upon retargeting of the AdV vector to either EpCAM or EGFR, respectively. Cells were labeled with cell-permeant non-fluorescent CellTrace dyes, which react with amino groups and become fluorescent. Yellow, population one of the tumor cells (P1, labeled cell population) transduced with AdV in the respective samples; cyan, nuclei (Hoechst, both populations); magenta, IgA (detected by goat anti-human kappa light chain); green, SIRPα-Fc (detected by goat anti-human IgG Fc). The merged images (cyan and yellow) show the overlay between the nuclei (both populations) and the transduced cells (P1). Staining of IgA or SIRPα-Fc in non-yellow areas indicates binding to non-transduced cells in a paracrine fashion. Scale bar: 100 µm. Please note that control panels for MDA-MB-468 (labeled NT) are also utilized in Appendix Fig. S5. (**C**) A close-up of MDA-MB-468 cells producing IgA and SIRPα-Fc in a paracrine fashion. Cells were not permeabilized before staining. NT, non-treated. Scale bar: 50 µm. Positive controls (purified recombinant IgA, SIRPα-Fc proteins) and secondary Ab-only control images are shown in Appendix Fig. S5. MOI: 20 i.v.p./cell (each virus). Representative images from three biological replicates are shown.

 © The Author(s)

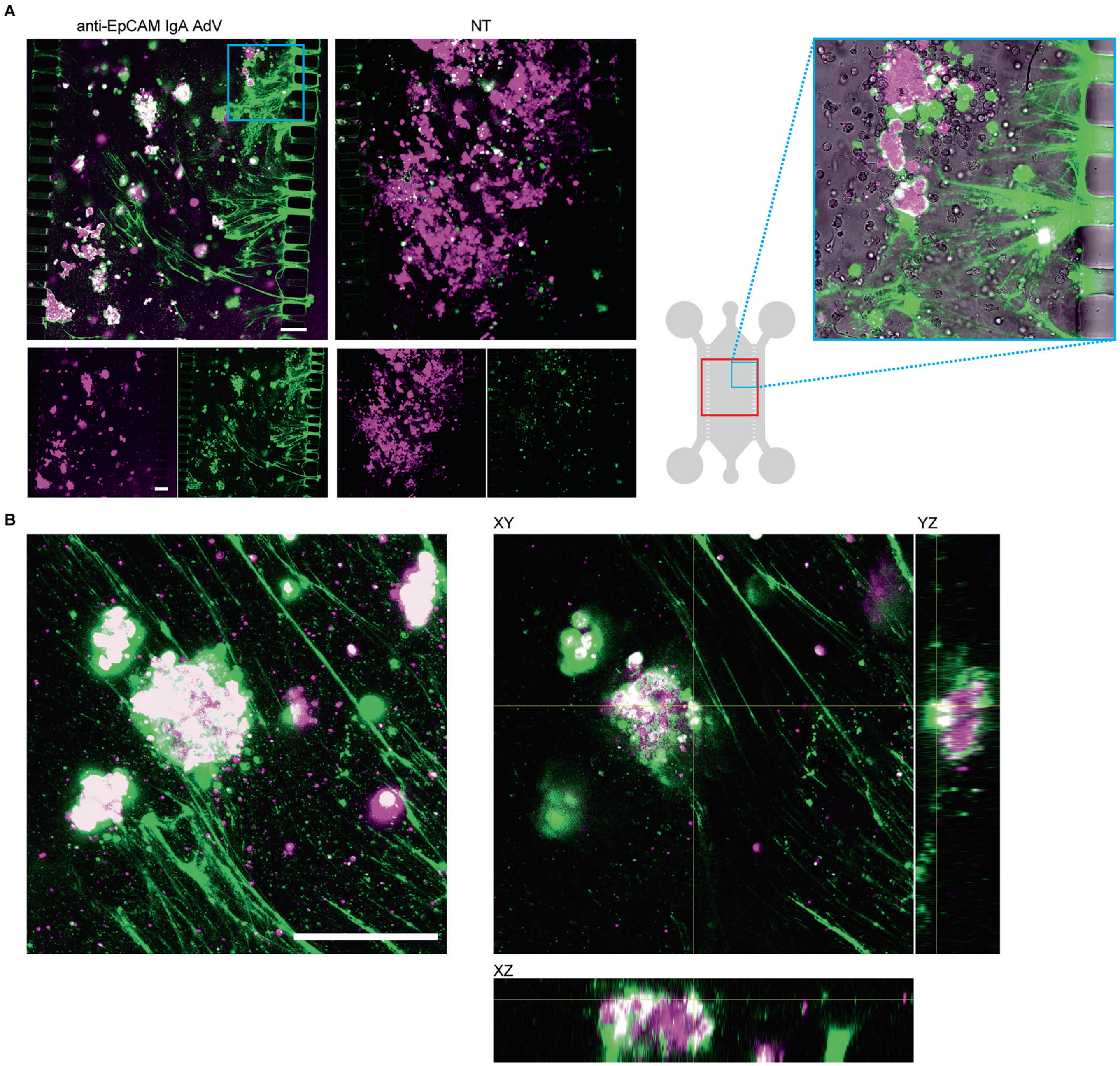

Figure EV4. Neutrophil extracellular traps (NETs) were detected on-chip following AdV-mediated local IgA production, triggering neutrophil ADCC.

(**A**) Neutrophil activity is seen on-chip where anti-EpCAM IgA was delivered in the HCT116 model (left), versus non-treated (NT) control. Magenta: HCT116 cancer cells (CellTrace Far Red), green: DNA, non-viable cells (CellTox Green). Overlay images are shown in which co-localization of both signals indicates dead cancer cells; cells with only green signal represent non-viable neutrophils. Filamentous structures in the adenovirus-treated sample are likely NETs, which consist mostly of DNA, released by neutrophils that were perfused on-chip through the right-side channel. The schematic image represents the tumor-on-a-chip (not drawn to scale), with the approximate areas in the microscopy images shown in red; inset: a close-up of the tissue compartment showing the tunnel connections through which neutrophils infiltrate from the side channel. Cropped areas of the upper left panel are also utilized in Appendix Fig. S7C for an illustrative example of the ADCC quantification approach (middle and bottom image rows). (**B**) A 3D reconstruction of cancer cells being eliminated by neutrophil ADCC, represented as a maximum intensity projection (left) and orthogonal sections (right). Representative images are shown, $n = 3$ biological replicates. Scale bar: 100 µm.

© The Author(s)

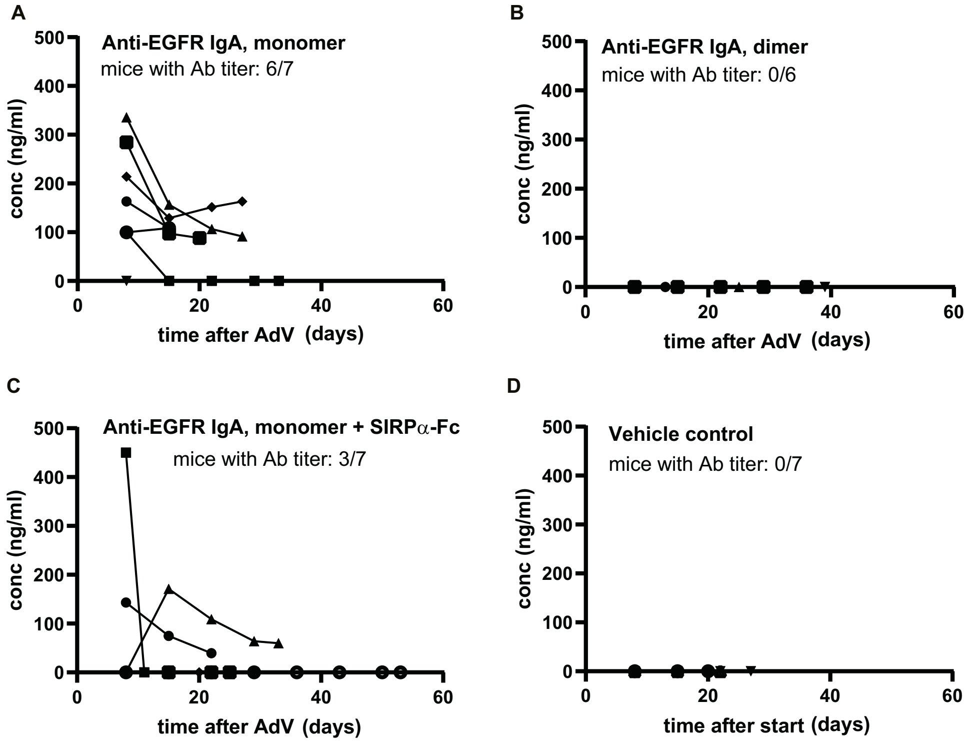

Figure EV5. IgA antibody titers from MDA-MB 468 tumor-bearing SCID FcαRI transgenic mice injected with anti-EGFR IgA- and SIRPα-Fc-encoding adenoviral vectors.

IgA titers in plasma samples from tumor-bearing mice were measured by ELISA using monomeric IgA as a standard, with anti-human kappa light chain used for coating and anti-human IgA-HRP used for detection. Anti-EGFR IgA levels were detected upon treatment with (**A**) monomeric anti-EGFR IgA-encoding adenoviral vectors, (**B**) dimeric anti-EGFR IgA-encoding adenoviral vectors, (**C**) monomeric anti-EGFR IgA and SIRPα-Fc-encoding adenoviral vectors, or (**D**) vehicle control. Each symbol represents a different mouse.

 © The Author(s)
