# Supplementary material for: Retargeted adenoviruses for local IgA and CD47 blocker production as a novel cancer therapy
Source: EMBO Mol Med. 2026 Jul 7;18(8):3226–57. doi: 10.1038/s44321-026-00477-7 (PMC13469128; doi:10.1038/s44321-026-00477-7)
Supplement: Supplementary file 2 [file 44321_2026_477_MOESM1_ESM.pdf]

Appendix for

Retargeted adenoviruses for local IgA and CD47 blocker production as a novel cancer therapy

Mariya Chernyavska et al.

Corresponding author: Dr. Wouter Verdurmen; Email: Wouter.verdurmen@radboudumc.nl

Table of content

Appendix Figure S1: Expression of IgA antibodies from P2A and T2A vector variants compared in ELISA……..…2

Appendix Figure S2: Immunofluorescence-based confirmation of IgA antibody and SIRPα-Fc production in CHO-S cells through target binding in cancer cell lines…………………………………………….….3

Appendix Figure S3: Assessment of the ability of the purified IgA antibodies to induce antibody-dependent cell-mediated cytotoxicity (ADCC) of tumor cells………………………………………………..…4

Appendix Figure S4: Immunofluorescence-based detection of IgA and SIRPα-Fc recombinant controls in HCT116 and MDA-MB-468 cell lines……………………………………………………………...5

Appendix Figure S5: IgA and SIRPα-Fc co-delivered to tumor cells…………………………………………………6

Appendix Figure S6: IgA and SIRPα-Fc co-delivered to the tumor microenvironment on-chip……………….……7

Appendix Figure S7: IgA antibody-activated primary human neutrophils kill cancer cells in monolayer and tumor-on-a-chip models………………………………………………………………………….8

Appendix Figure S8: ADCC upon retargeting of adenoviral vector encoding IgA or SIRPα-Fc to cancer cells via EGFR or EpCAM in the tumor-on-a-chip……………………………………………...…10

Appendix Figure S9: Effect of SIRPα-Fc protein on recombinant IgA-mediated neutrophil ADCC on-chip……….11

Appendix Figure S10: Immunofluorescence analysis of MDA-MB-468 tumor slices from SCID mice injected with PBS……………………………………………………………………………………12

Appendix Figure S11: Immunofluorescence analysis of HCT116 tumor slices from SCID mice injected with anti-EpCAM IgA- and SIRPα-Fc-encoding adenoviral vectors……………………………………13

Appendix Figure S12: Immunofluorescence analysis of HCT116 tumor slices from SCID mice injected with PBS as control……………………………………………………………………………………14

Appendix Figure S13: Immunofluorescence analysis of B16F10 tumors from C57BL/6 mice injected with anti-EGFR IgA- and SIRPα-Fc-encoding adenoviral vectors……………………………………………..15

Appendix Figure S14: Immunofluorescence analysis of B16F10 tumors from C57BL/6 mice injected with PBS as control……..………………………………………………………………………...……16

Appendix Figure S15: IgA titers from HCT116 tumor-bearing SCID FcαRI transgenic mice injected with anti-EpCAM IgA- and SIRPα-Fc-encoding adenoviral vectors………........……………….…..17

Appendix Figure S16: IgA titers from B16F10 tumor-bearing C57BL/6 FcαRI transgenic mice injected with anti-EGFR IgA- and SIRPα-Fc-encoding adenoviral vectors…..……………………………...18

Appendix Figure S17: Gene expression analysis of B16F10 tumors extracted from C57BL/6 FcαRI transgenic mice.19

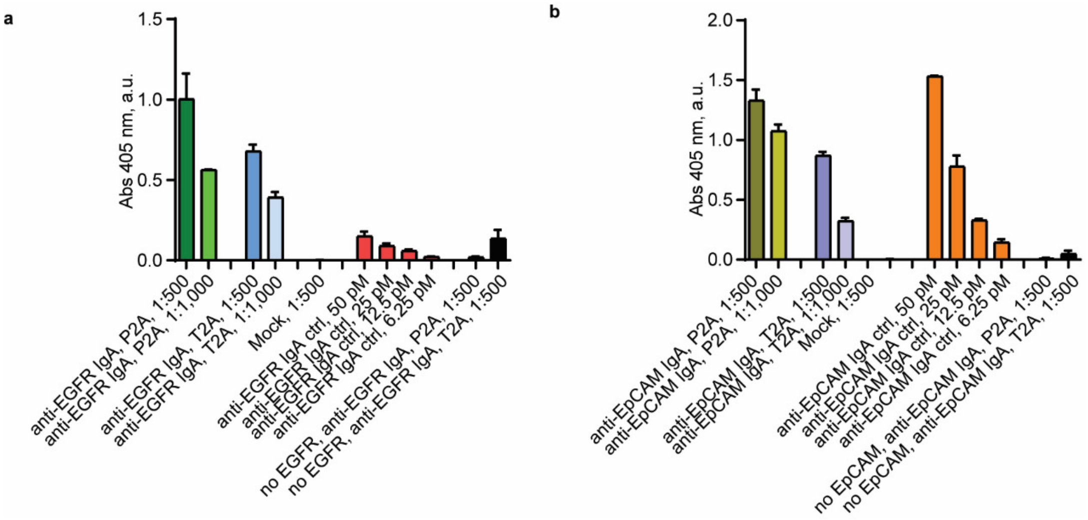

Appendix Figure S1. Expression of IgA antibodies from P2A and T2A vector variants compared in ELISA. **(a)** ELISA-based detection of anti-EGFR IgA in supernatants (at 1:500 and 1:1,000 dilution) of CHO-S cells transfected with the IgA-encoding constructs, containing either P2A or T2A "self-cleaving" (ribosome-skipping) sequences. Mock-transfected (eGFP construct) CHO-S supernatant was used as a negative control. Recombinant anti-EGFR IgA produced by co-expression of heavy and light chains in trans was used as a positive control. To evaluate non-specific binding, a control without coated EGFR domains was included. **(b)** Same as (a), for anti-EpCAM IgA. Representative data from three independent experiments is shown (mean ± range from two technical replicates indicated).

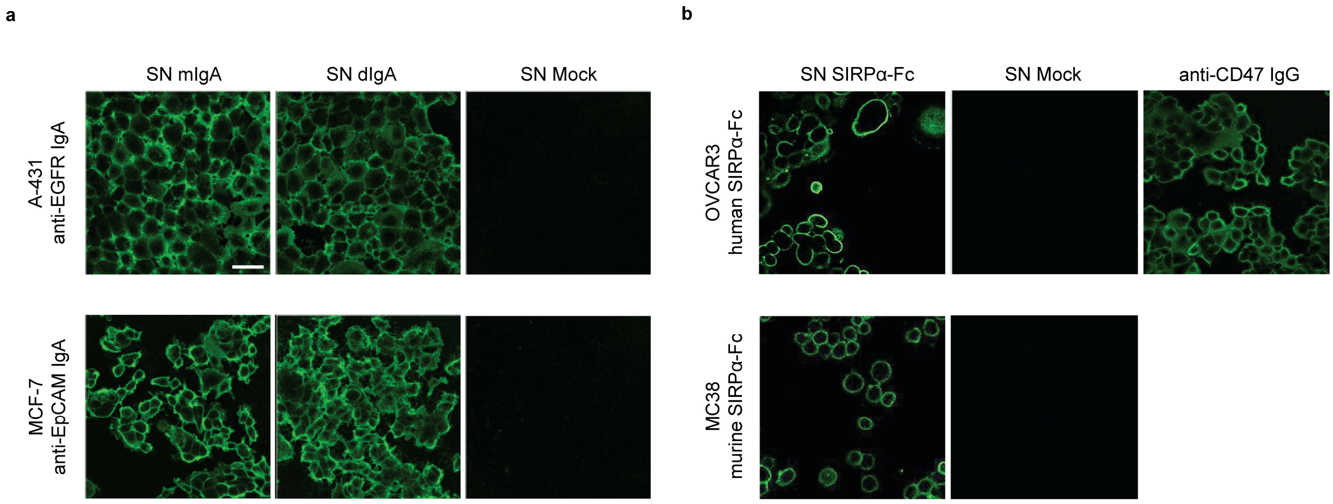

Appendix Figure S2. Immunofluorescence-based confirmation of IgA antibody and SIRPα-Fc production in CHO-S cells through target binding in cancer cell lines. Upon transfection of CHO-S cells with the respective expression vectors, the supernatants (SN) containing the molecule of interest were incubated with the tumor cells expressing the respective target surface receptor, and the binding of the target molecule to the cell surface was visualized by indirect immunofluorescence. **(a)** Supernatants from CHO-S cells transfected with the expression constructs for anti-EGFR or anti-EpCAM IgA monomer (mIgA) or dimer (dIgA), as well as mock-transfected CHO-S supernatants (GFP expression construct) were analyzed by incubating the SN with EGFR-overexpressing A-431 cells, or EpCAM-overexpressing MCF-7 cells, respectively. Note that the dimer-encoding vector produces a combination of monomer and dimer and that we stain for IgA irrespective of its multimerization state. **(b)** Supernatants from CHO-S cells transfected with the SIRPα-Fc-expressing construct or GFP-expressing construct (SN Mock) were analyzed by incubating the SN with OVCAR3 cells (expressing human CD47) or MC38 cells (expressing murine CD47). Positive control: anti-human CD47 IgG. The representative images from three independent experiments are shown. Scale bar: 100 µm.

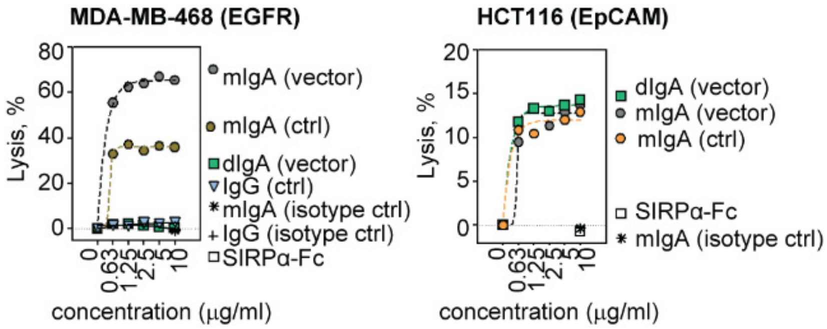

Appendix Figure S3. Assessment of the ability of the purified IgA antibodies to induce antibody-dependent cell-mediated cytotoxicity (ADCC) of tumor cells. MDA-MB-468 and HCT116 cells were used as target cells for anti-EGFR and anti-EpCAM IgA-mediated killing by primary human neutrophils, respectively. IgA/IgG ctrl refers to pure proteins produced using conventional approaches as opposed to purified from supernatants of adenoviral vector-transduced cells. For MDA-MB-468 cells, IgG was used as a negative control for the assay. Of note, for the purified dimeric mix of anti-EGFR IgA, we observed aggregation and a complete lack of cell-killing activity. N=3, a representative experiment is shown.

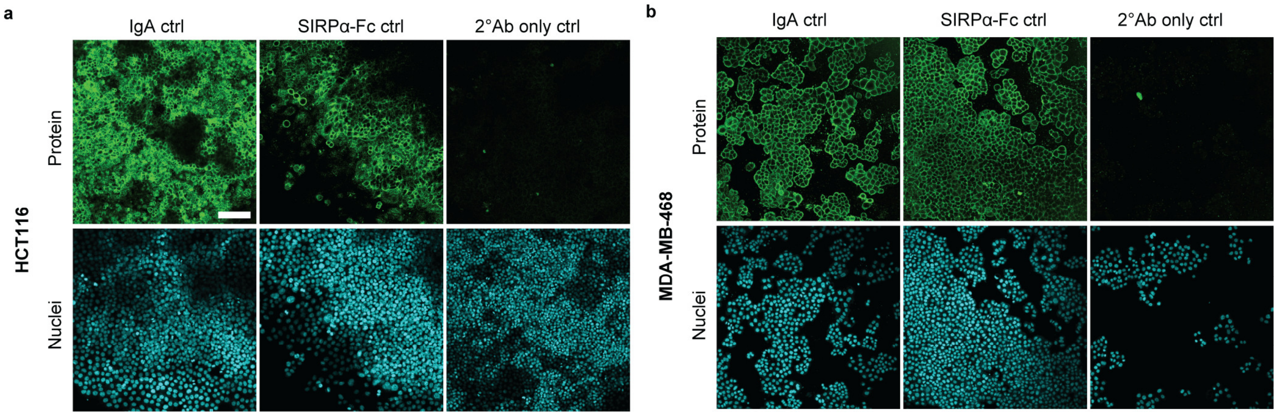

Appendix Figure S4. Immunofluorescence-based detection of IgA and SIRPα-Fc recombinant controls in HCT116 and MDA-MB-468 cell lines. **(a)** Binding of anti-EpCAM IgA and SIRPα-Fc recombinant proteins at 20 nM was detected by using goat-anti-human kappa light chain and goat anti-human IgG Fc as primary antibodies, respectively. Staining with a secondary rabbit anti-goat antibody only (2º Ab only ctrl) was performed as a negative control for both IgA and SIRPα-Fc. **(b)** Same as (a), for anti-EGFR IgA and SIRPα-Fc recombinant proteins in MDA-MB-468 cells. Scale bar: 100 µm. Please note that control panels for 2º Ab only MDA-MB-468 ctrl are reused from Figure 2C.

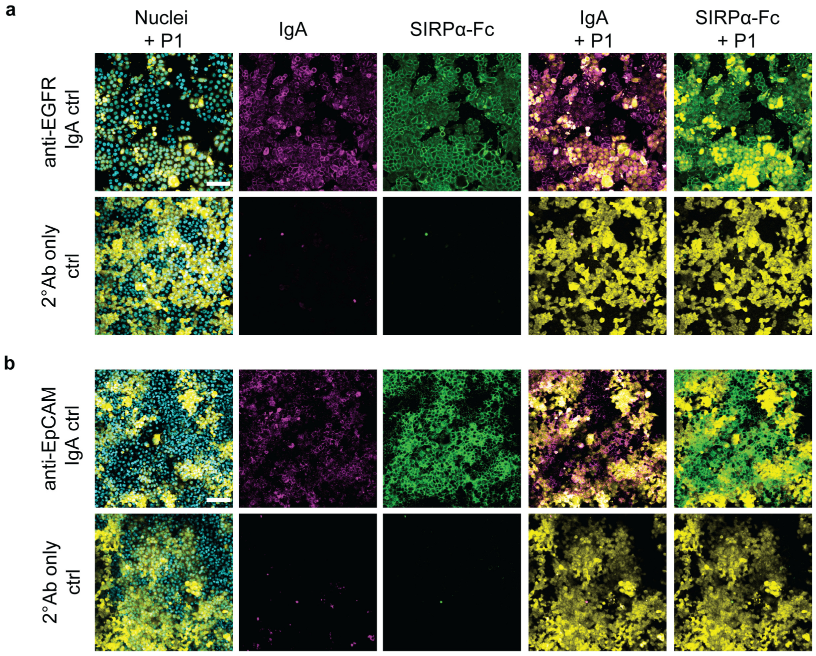

Appendix Figure S5. IgA and SIRPα-Fc co-delivered to tumor cells. (a) MDA-MB-468 cells were seeded into the same well in two populations, one population labeled with CellTrace Yellow (P1), and one non-labeled. Nuclei of both populations were stained with Hoechst (cyan). As a positive control for co-delivery of IgA and SIRPα-Fc (Figure EV3), the recombinant proteins at 20 nM each were added to the tumor cells and detected simultaneously in indirect immunofluorescence. Magenta: anti-EGFR IgA; green: SIRPα-Fc. Similar to Figure EV3, the overlay images of IgA or SIRPα-Fc with the labeled tumor cell population are shown. Please note that control panels for MDA-MB-468 (labeled 2º Ab only) are also utilized in Figure EV3A. **(b)** Same as (a), for HCT116 cells, and anti-EpCAM IgA. Scale bar: 100 µm.

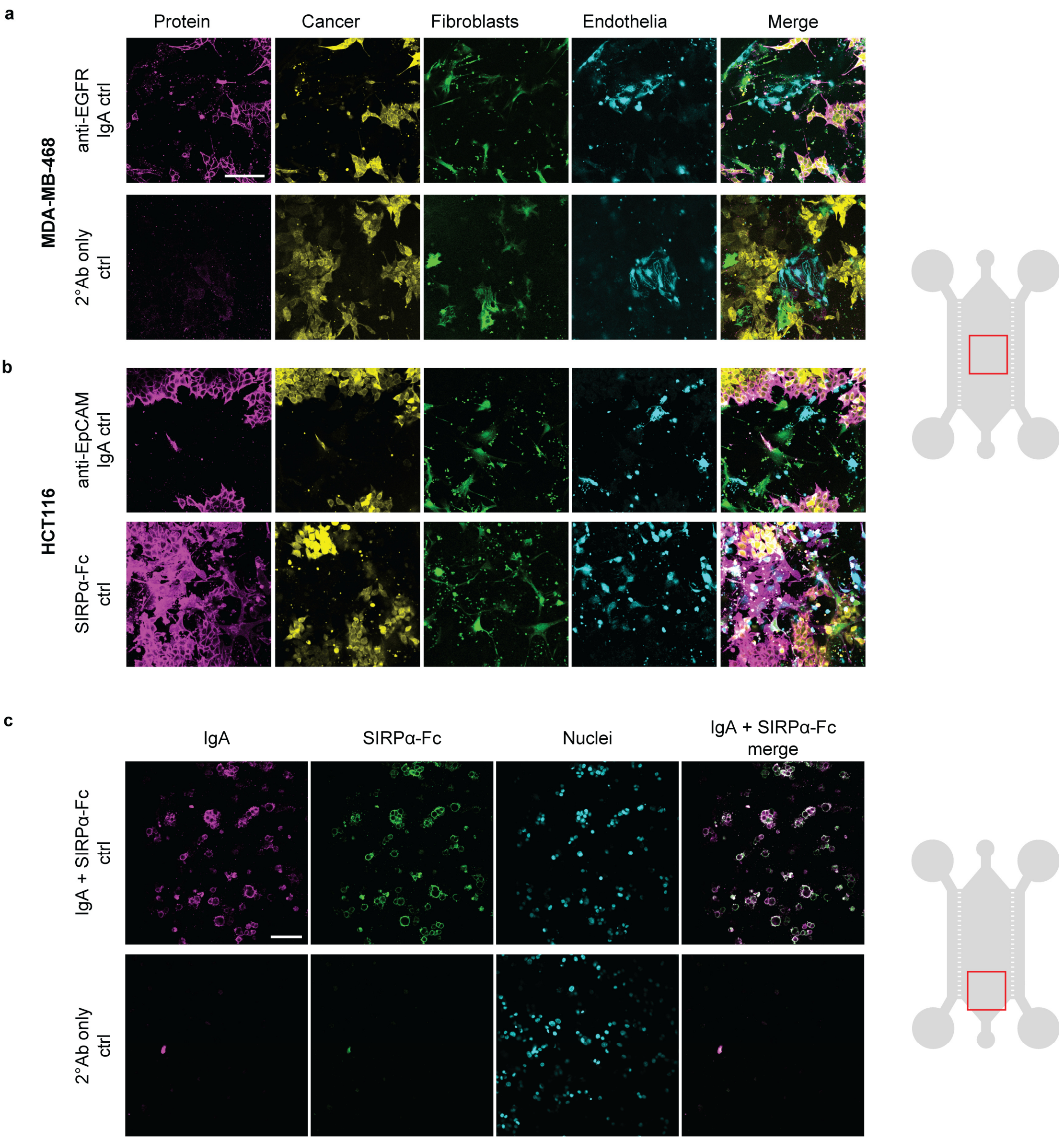

Appendix Figure S6. IgA and SIRPα-Fc co-delivered to the tumor microenvironment on-chip. (a) Anti-EGFR IgA (magenta) was added at 20 nM as a recombinant protein control; secondary antibody only (2ºAb only ctrl) is shown. As in Figure 3, the schematic image represents the tumor-on-a-chip with an approximate image area indicated. Yellow (CellTrace Far Red-stained): MDA-MB-468 cells; green (Cell Trace yellow-stained): fibroblasts; cyan (CellTrace Violet-stained): endothelial cells; overlay images demonstrate the specificity of IgA binding on the surface of the cancer cells. **(b)** Recombinant controls for anti-EpCAM IgA and SIRPα-Fc (magenta) in HCT116 tumor-on-a-chip model, similar to (a). Anti-EpCAM IgA showed specificity for cancer cells, whereas SIRPα-Fc was detected on the surface of all cell types. (**c**) Positive control and secondary antibody only control for co-delivery in Figure 3e. Magenta: recombinant anti-EGFR IgA (20 nM); green: recombinant SIRPα-Fc (20 nM); cyan: nuclei. Similar to (a) and (b), the tumor-on-a-chip schematic image shows the approximate area imaged. Representative images are shown from at least two chips in at least two independent experiments. Scale bar: 100 µm.

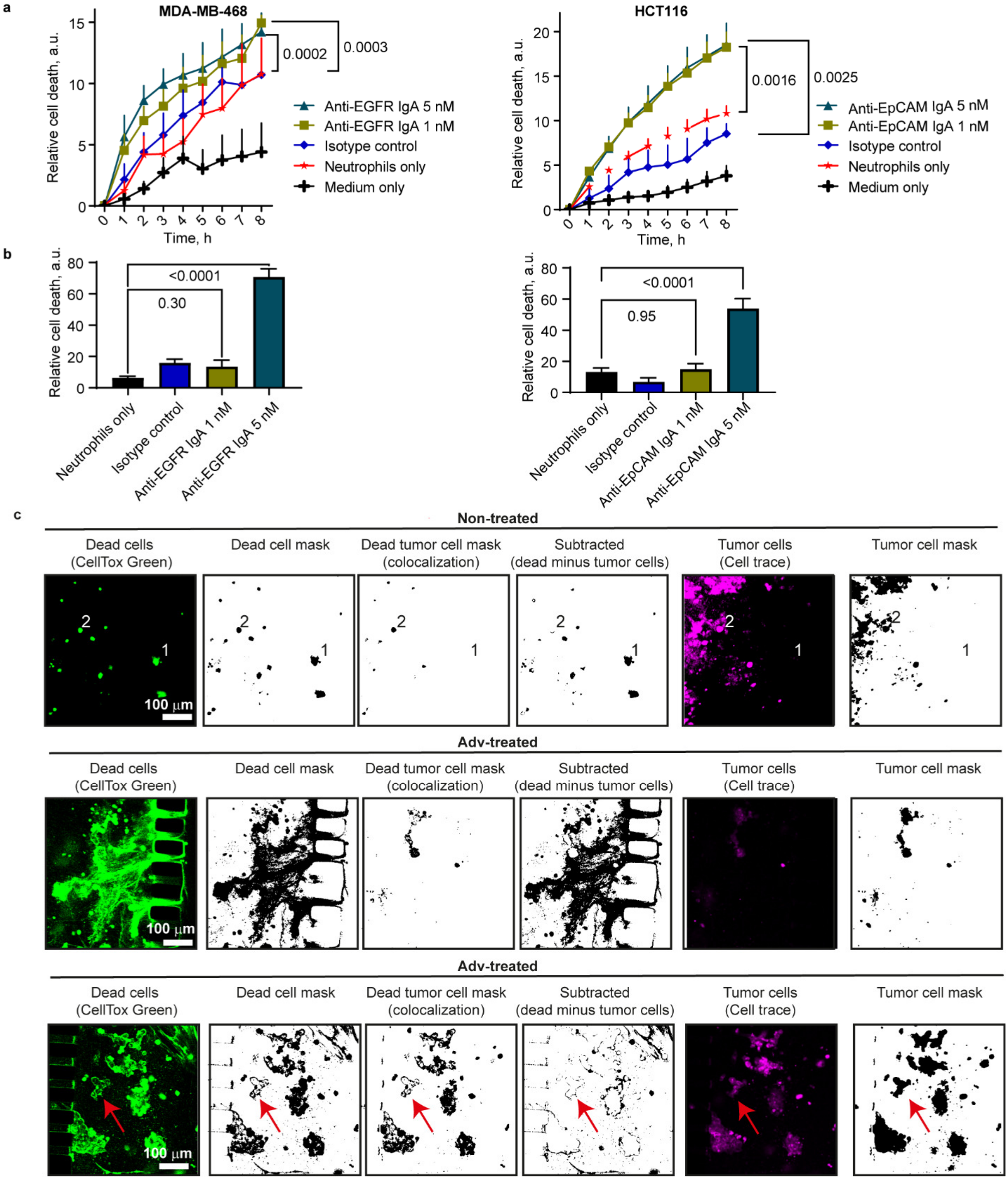

Appendix Figure S7. IgA antibody-activated primary human neutrophils kill cancer cells in monolayer and tumor-on-a-chip models. (a) Time course of image-based ADCC analysis in monolayer models mediated via anti-EGFR IgA in MDA-MB-468, and via anti-EpCAM IgA in HCT116 cells. Recombinant IgA was used to assess the activity of the neutrophils co-cultured with tumor cells. As negative controls, a non-binding IgA (isotype control), or no antibody (neutrophils only) or no effector cells (medium only) were used. Each time point is shown as mean with SEM from four independent experiments, with effector cells from four different blood donors. Statistical analysis: one-way ANOVA with Dunnet's correction for multiple comparisons, adjusted p-values are shown. **(b)** ADCC in

tumor-on-a-chip models (left, MDA-MB-468; right, HCT116). Anti-EGFR IgA and anti-EpCAM IgA were tested at the same concentrations as in (a), and neutrophils only (without antibody) or isotype control were used as negative controls. The values were normalized for the total sum per experiment and are shown as mean and SEM, for at least n=5 biological replicates (each value was obtained as an average from analyzing two representative chip areas, covering approx. 30% of the tissue compartment each), from three different blood donors. Statistical analysis: one-way ANOVA with Dunnet's correction for multiple comparisons, adjusted p-values are shown. **(c)** Top: Illustrative example of step-wise masking approach for non-treated HCT116 cells, which is part of the ADCC quantification approach. Dead cells are seen that do not show overlap with tumor cells (# 1: no overlap of 'Tumor cell mask' with 'Dead cell mask'), and which likely reflect dead neutrophils. Some dead tumor cells are still seen (# 2: 'Tumor cell mask overlaps with 'Dead cell mask'). Middle: Illustrative example of Anti EpCAM IgA AdV-treated HCT116 cells (targeted to EGFR), where a region is shown with high CellTox green signal but low tumor cell signal, indicating many dead neutrophils. Since only the colocalization counts toward tumor cell killing, the large amount of CellTox green signal does not negatively affect the quantification analyses, which are based on the colocalization signal. Bottom: Example of wrapping DNA around live tumor cells and associated analyses, for HCT116 cells treated with anti-EpCAM IgA-encoding AdV (targeted to EGFR). The red arrow illustrates an example where live tumor cells are wrapped with DNA (CellTox green staining). The dead cell mask clearly shows that only the outer rim is considered positive, but that the core area shows no overlap, indicating limited bias. The middle and bottom examples correspond to cropped areas of the upper left panel of Fig. EV4A, where an overlay of tumor cell mask and CellTox green is shown.

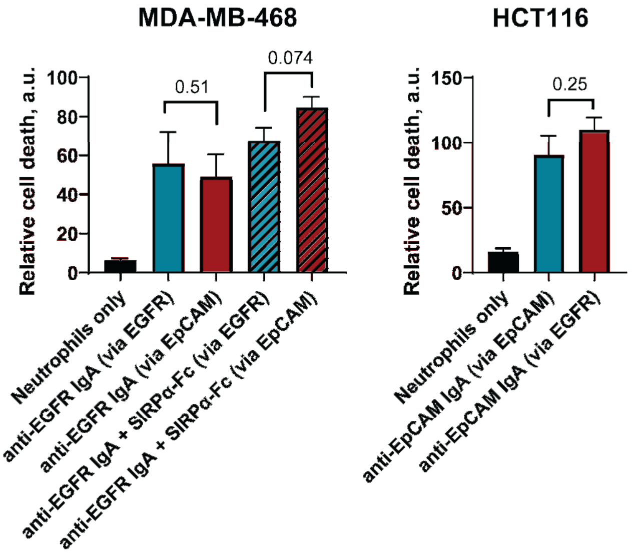

Appendix Figure S8. ADCC upon retargeting of adenoviral vector encoding IgA or SIRPα-Fc to cancer cells via EGFR or EpCAM in the tumor-on-a-chip. ADCC activity was measured by an image-based assay (see Methods). In MDA-MB-468, ADCC was mediated via anti-EGFR IgA, and in HCT116, ADCC was mediated via anti-EpCAM IgA. As both EpCAM and EGFR could be targeted with adenovirus for the IgA (and SIRPα-Fc) gene delivery, both retargeting strategies were compared. Neutrophils without antibody served as a control. The values were normalized based on the sum of the values of each experiment and are shown as mean and SEM, for at least n=5 biological replicates (each value was obtained as an average from analyzing two representative chip areas, covering approx. 30% of the tissue compartment each), from three different blood donors. Statistical analysis: unpaired t-test, adjusted p-values are shown.

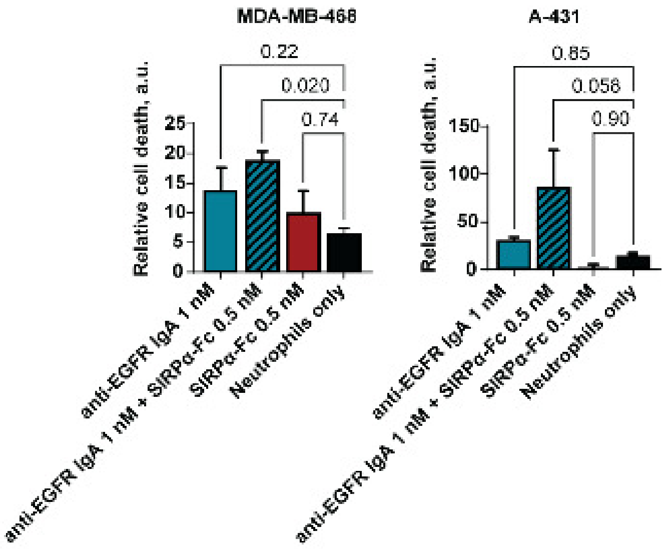

Appendix Figure S9. Effect of SIRPα-Fc protein on recombinant IgA-mediated neutrophil ADCC on-chip. The effects of CD47 blockade were tested in MDA-MB-468 and A-431 cell lines, alone or in combination with anti-EGFR IgA. Neutrophils only: neutrophils added to the cancer cells without treatment. The values were normalized for the total sum of relative cell death values per experiment and are shown as mean ± SEM, for n=5 chips (MDA-MB-468), and n=2 chips (A-431). Each value was obtained as an average from analyzing two representative chip areas, covering approx. 30% of the tissue compartment each. Statistical analysis: one-way ANOVA with Dunnet's correction for multiple comparisons, adjusted p-values shown.

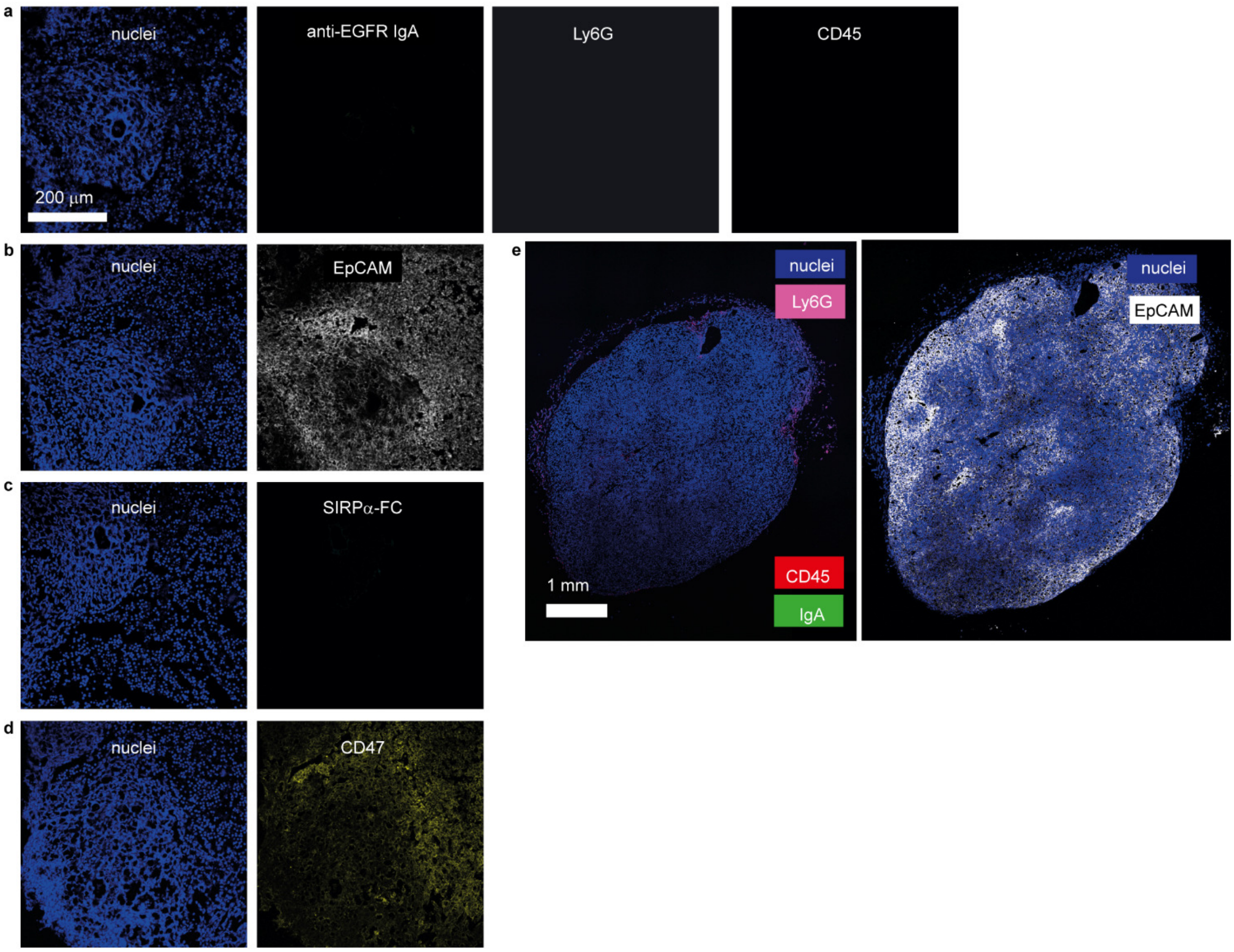

Appendix Figure S10. Immunofluorescence analysis of MDA-MB-468 tumor slices from SCID mice injected with PBS. (a) Immunofluorescence (IF) of tumor slices from MDA-MB-468 tumor-bearing SCID mice four days after injection with PBS. Nuclei were stained with DAPI. Ly6G and CD45 staining was detected in the same slice indicating the presence of neutrophils and immune cells, respectively. **(b-d)** Detection of EpCAM **(b)**, SIRPα-Fc **(c)** and CD47 **(d)** by IF as in (a). **(e)** A tile scan of a section of the entire tumor slice was analyzed by IF with the conditions described in (a) (left) or stained with anti-EpCAM IgA (right). The left image shows very little immune cell infiltration. The scale bar in (a) is applicable to all images from a-d. n = 4, representative images are shown.

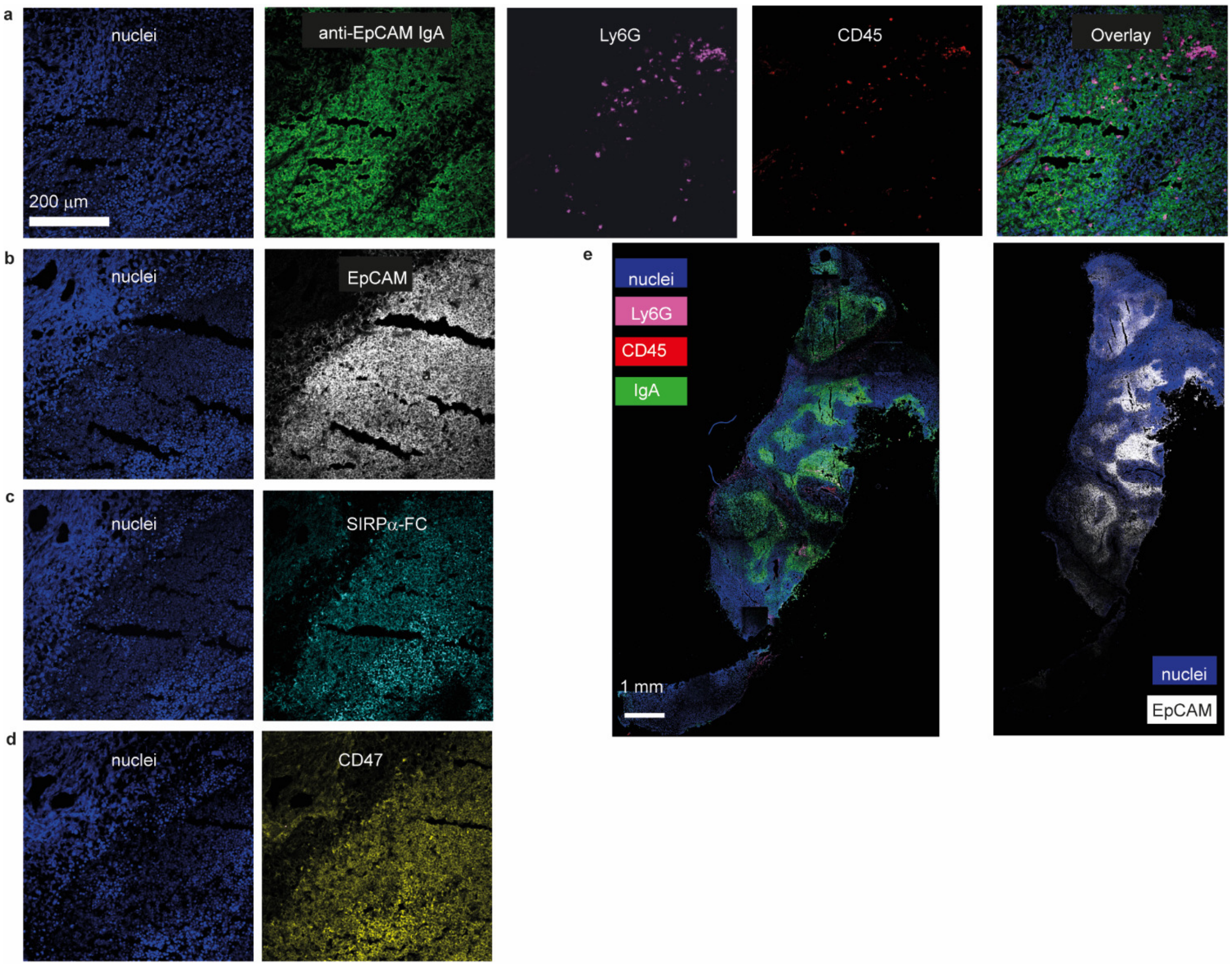

Appendix Figure S11. Immunofluorescence analysis of HCT116 tumor slices from SCID mice injected with anti-EpCAM IgA- and SIRPα-Fc-encoding adenoviral vectors. (a) Immunofluorescence (IF) of tumor slices from HCT116 tumor-bearing SCID mice four days after injection with EGFR-directed anti-EpCAM IgA-encoding and SIRPα-Fc-encoding adenoviral vectors. Nuclei were stained with DAPI. Ly6G and CD45 were detected in the same slice as markers for neutrophils and immune cells, respectively. **(b-d)** Detection of EpCAM **(b)**, SIRPα-Fc **(c)** and CD47 **(d)** by IF as in (a). **(e)** A tile scan of a section of the entire tumor slice was analyzed by IF for the conditions described in (a) (left), or anti-EpCAM IgA (right). The scale bar in (a) is applicable to all images from a-d. n = 6, representative images are shown.

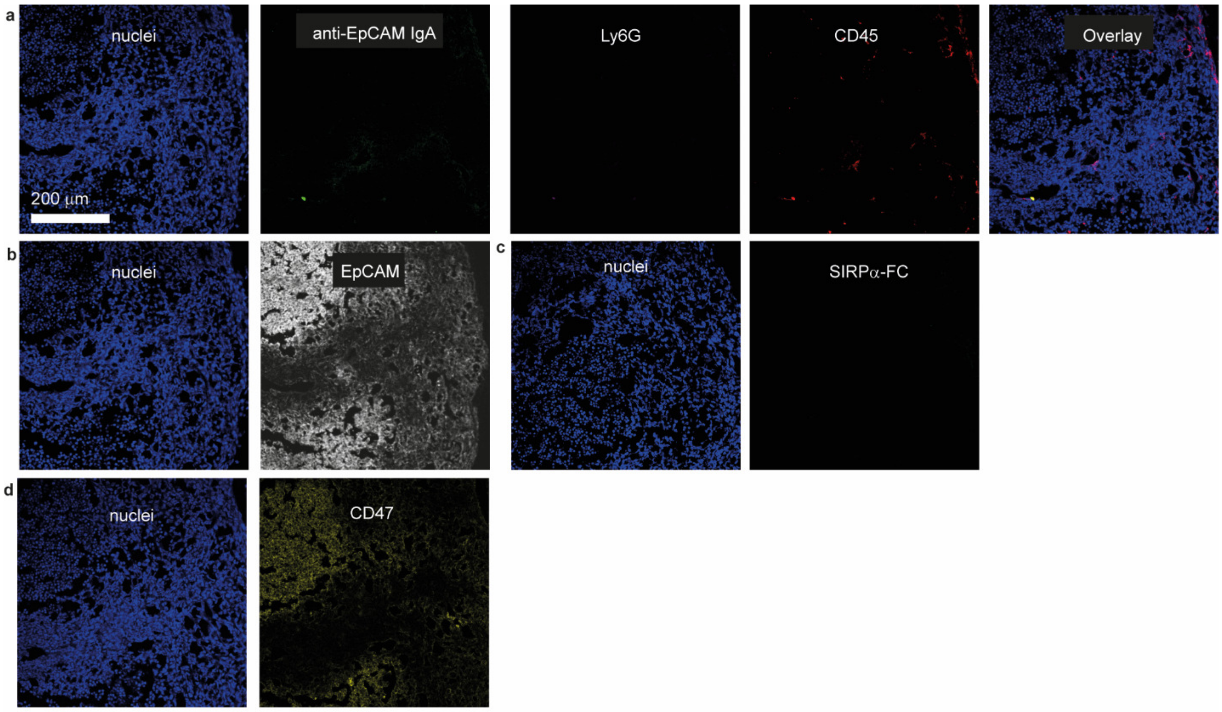

Appendix Figure S12. Immunofluorescence analysis of HCT116 tumor slices from SCID mice injected with PBS as control. (a) Immunofluorescence (IF) of tumor slices from HCT116 tumor-bearing SCID mice four days after injection with PBS. Nuclei were stained with DAPI. Ly6G and CD45 were detected in the same slice as markers for neutrophils and immune cells, respectively. **(b-d)** Detection of EpCAM **(b)**, SIRPα-Fc **(c)** and CD47 **(d)** by IF as in (a). The scale bar in (a) is applicable to all images. n = 6, representative images are shown.

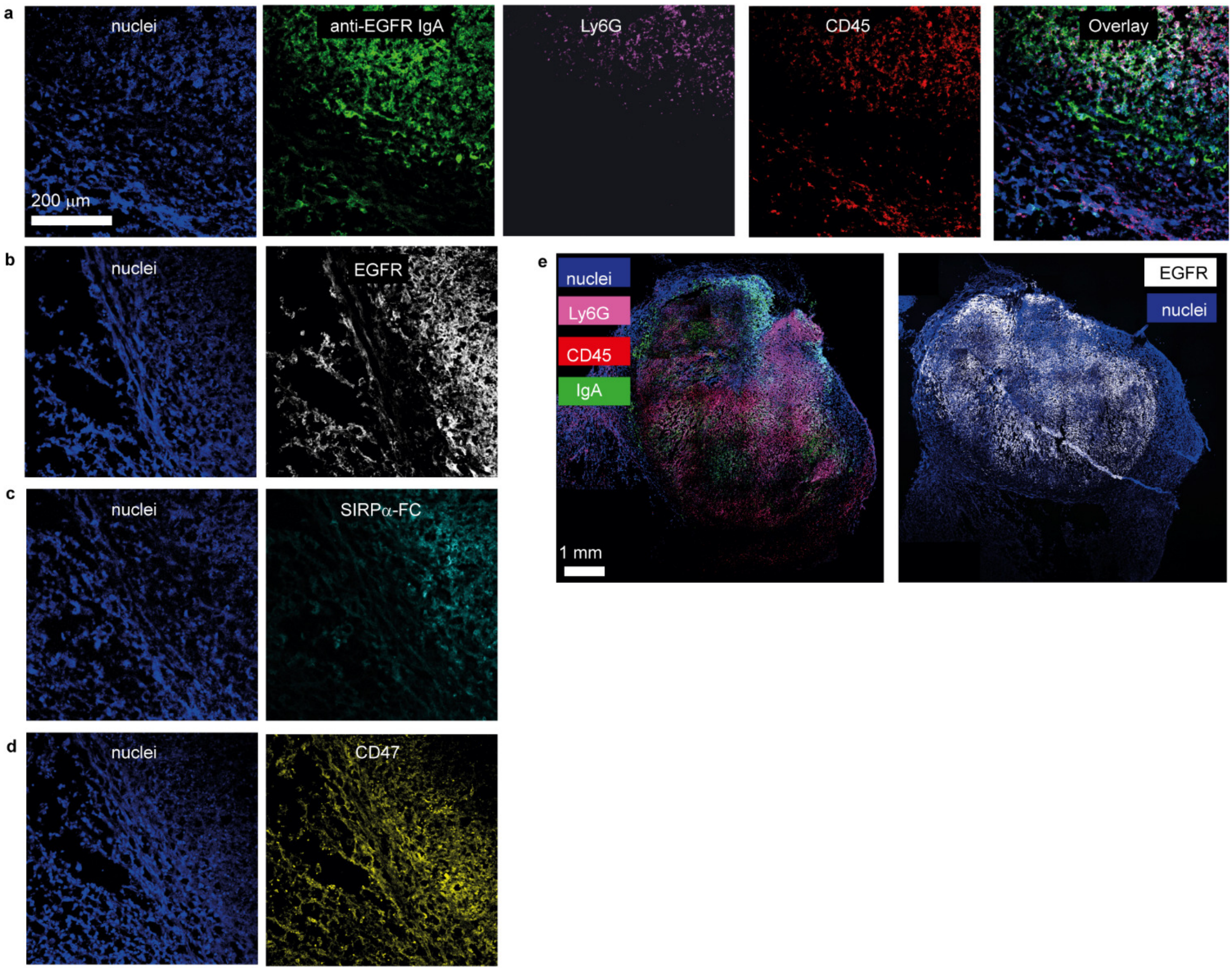

Appendix Figure S13. Immunofluorescence analysis of B16F10 tumors from C57BL/6 mice injected with anti-EGFR IgA- and SIRPα-Fc-encoding adenoviral vectors. **(a)** Immunofluorescence (IF) of tumor slices from B16F10 tumor-bearing C57Bl/6 mice four days after injection with EGFR-directed anti-EGFR IgA-encoding and SIRPα-Fc-encoding adenoviral vectors. Nuclei were stained with DAPI. Ly6G and CD45 were detected in the same slice as markers for neutrophils and immune cells, respectively. **(b-d)** Detection of EGFR **(b)**, SIRPα-Fc **(c)** and CD47 **(d)** by IF as in (a). **(e)** A tile scan of a section of the entire tumor slice was analyzed by IF for the condition described in (a) (left), or EGFR (right). The scale bar in (a) is applicable to all images from a-d. n = 6, representative images are shown.

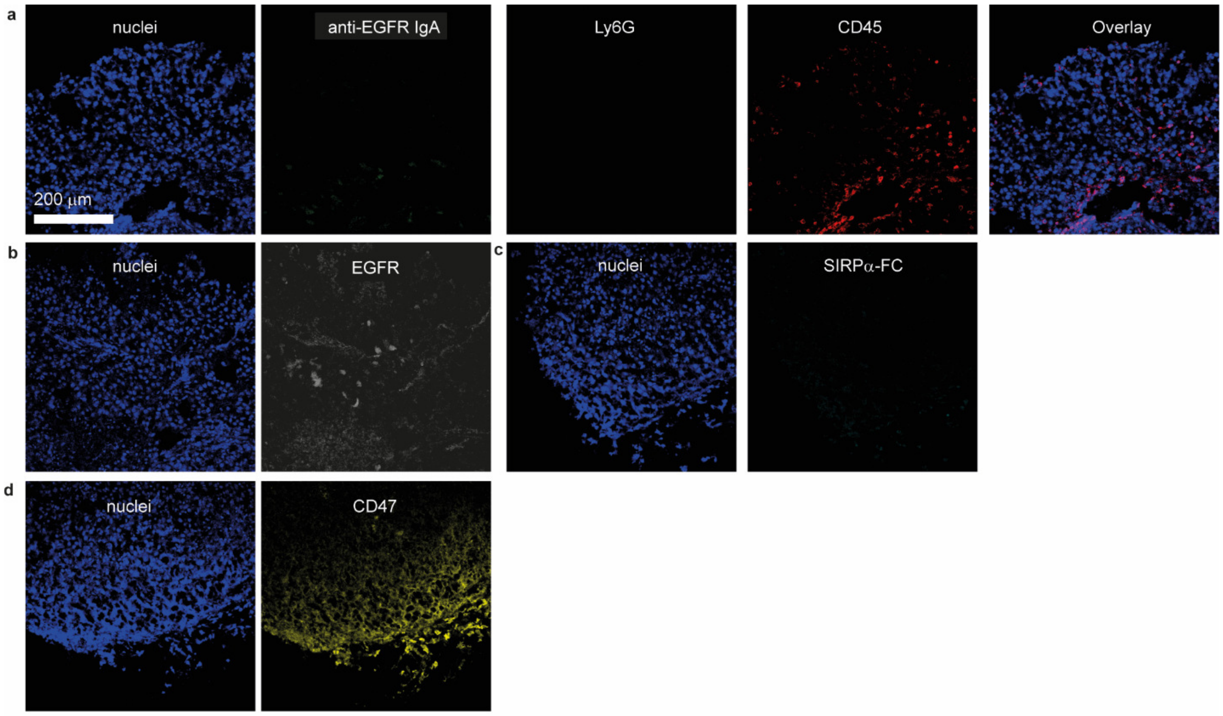

Appendix Figure S14. Immunofluorescence analysis of B16F10 tumors from C57BL/6 mice injected with PBS as control. (a) Immunofluorescence (IF) of tumor slices from B16F10 tumor-bearing C57Bl/6 mice four days after injection with PBS. Nuclei were stained with DAPI. Ly6G and CD45 were detected in the same slice as markers for neutrophils and immune cells, respectively. **(b-d)** Detection of EGFR **(b)**, SIRPα-Fc **(c)** and CD47 **(d)** by IF as in (a). The scale bar in (a) is applicable to all images. n = 6, representative images are shown.

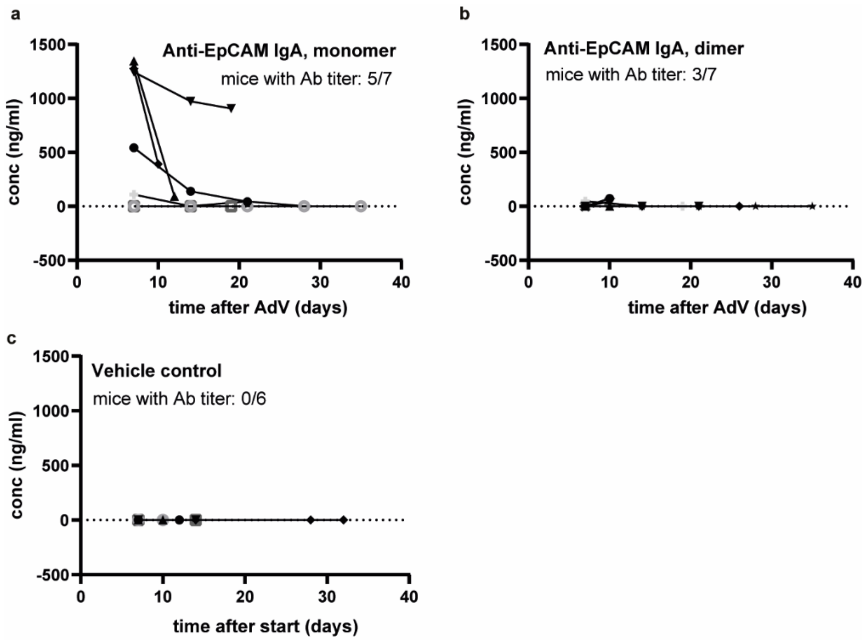

Appendix Figure S15. IgA titers from HCT116 tumor-bearing SCID FcαRI transgenic mice injected with anti-EpCAM IgA- and SIRPα-Fc-encoding adenoviral vectors. IgA titers in plasma samples from tumor-bearing mice were measured using ELISA using monomeric IgA as a standard, with anti-human kappa light chain used for coating and anti-human IgA-HRP used for detection. Anti-EpCAM IgA levels were detected upon treatment with (**a**) monomeric anti-EpCAM IgA-encoding adenoviral vectors, (**b**) dimeric anti-EpCAM IgA encoding adenoviral vectors or (**c**) vehicle control. Each symbol represents a different mouse.

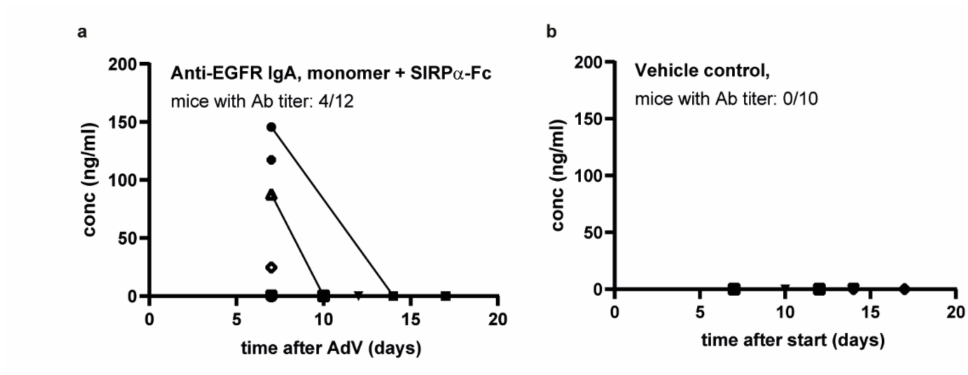

Appendix Figure S16. IgA titers from B16F10 tumor-bearing C57BL/6 FcαRI transgenic mice injected with anti-EGFR IgA- and SIRPα-Fc-encoding adenoviral vectors. IgA titers in plasma samples from tumor-bearing mice were measured using ELISA using monomeric IgA as a standard, with anti-human kappa light chain used for coating and anti-human IgA-HRP used for detection. Anti-EGFR IgA levels were detected upon treatment with (**a**) monomeric anti-EGFR IgA and SIRPα-Fc-encoding adenoviral vectors or (**b**) vehicle control. Each symbol represents a different mouse.

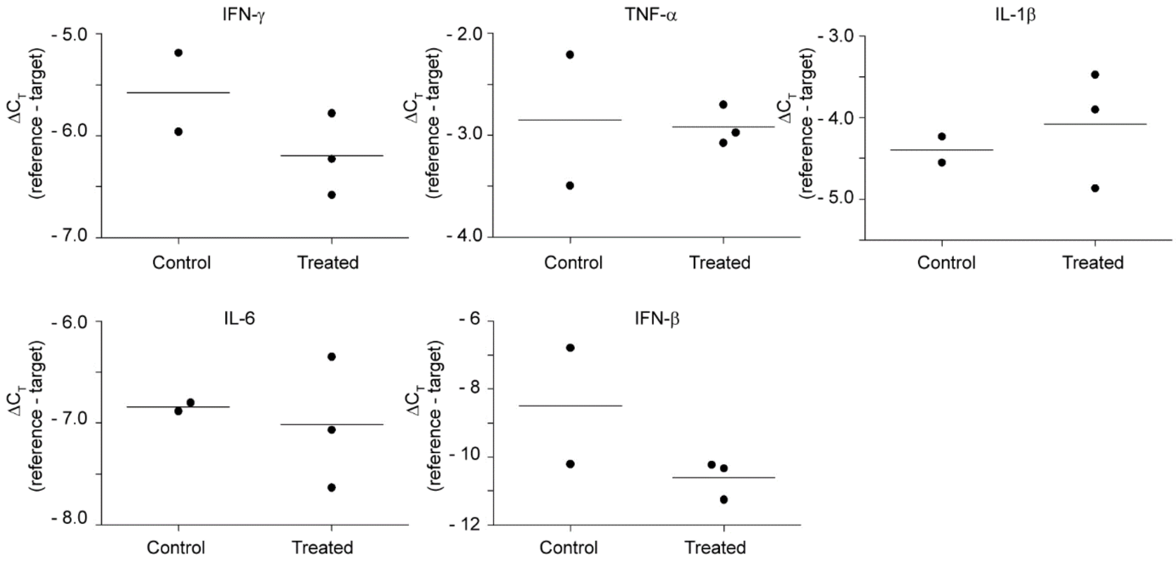

Appendix Figure S17. Gene expression analysis of B16F10 tumors extracted from C57BL/6 FcαRI transgenic mice. Gene expression of indicated immune-associated markers was determined using qPCR from tumors extracted at day 32 from mice that were vehicle treated or that were injected with anti-EGFR IgA- and SIRPα-Fc-encoding adenoviral vectors. Each dot represents a different mouse.
