# Supplementary material for: Retargeted adenoviruses for local IgA and CD47 blocker production as a novel cancer therapy
Source: EMBO Mol Med. 2026 Jul 7;18(8):3226–57. doi: 10.1038/s44321-026-00477-7 (PMC13469128; doi:10.1038/s44321-026-00477-7)
Supplement: Supplementary file 3 [file 44321_2026_477_MOESM2_ESM.pdf]

EMBO Molecular Medicine

Retargeted adenoviruses for local IgA and CD47 blocker production as a novel cancer therapy

Mariya Chernyavska, Karen Hartmann, J.H. Jansen, Niklas Baumann, Jonas Kolibius, Dominik Brücher, Theodora Kristoforus, Rens Peters, Lucas Huijs, Daphne Laarveld, Fabian Weiss, Renate Burger, Marta Lustig, Nadine Gimenez de Assis, Markus Schmid, Jeanette Leusen, Thomas Valerius, Andreas Plückthun, and Wouter Verdurmen

Corresponding author: Wouter Verdurmen (wouter.verdurmen@radboudumc.nl)

Review Timeline:

Submission Date:	19th Nov 25
Editorial Decision:	18th Dec 25
Revision Received:	13th Apr 26
Editorial Decision:	29th Apr 26
Revision Received:	9th Jun 26
Accepted:	16th Jun 26

Editor: Lise Roth

Transaction Report:

(Note: With the exception of the correction of typographical or spelling errors that could be a source of ambiguity, letters and reports are not edited. Depending on transfer agreements, referee reports obtained elsewhere may or may not be included in this compilation. Referee reports are anonymous unless the Referee chooses to sign their reports.)

18th Dec 2025

Dear Dr. Verdurmen,

Thank you for submitting your manuscript to EMBO Molecular Medicine. We have now received feedback from the two reviewers who agreed to evaluate your study. As you will see from the reports below, the reviewers acknowledge the potential interest and novelty of the findings but nevertheless raise a few major concerns. If you feel you can address the reviewers' comments satisfactorily, you may wish to submit a revised version of your manuscript.

Addressing the reviewers' concerns in full will be necessary for further considering the manuscript in our journal, and acceptance of the manuscript will entail a second round of review. EMBO Molecular Medicine encourages a single round of revision only and therefore, acceptance or rejection of the manuscript will depend on the completeness of your responses included in the next, final version of the manuscript. For this reason, and to save you from any frustrations in the end, I would strongly advise against returning an incomplete revision.

If you would like to discuss further the points raised by the referees, I am available to do so via email or video. Let me know if you are interested in this option.

We are expecting your revised manuscript within three months, if you anticipate any delay, please contact us.

When preparing your revised manuscript, please refer to our guidelines: https://link.springer.com/journal/44321/submission-guidelines#cms-Revised-submissions. We perform an initial quality control of all revised manuscripts before re-review; failure to include requested items will delay the evaluation of your revision.

We require:

1) A .docx formatted version of the manuscript text (including legends for main figures, EV figures and tables). Please make sure that the changes are highlighted to be clearly visible.

2) Individual production quality figure files as .eps, .tif, .jpg (one file per figure). For guidance, download the 'Figure Guide PDF': https://media.springernature.com/original/springer-cms/rest/v1/content/27825798/data/v1.

3) A .docx formatted letter INCLUDING the reviewers' reports and your detailed point-by-point responses to their comments. As part of the EMBO Press transparent editorial process, the point-by-point response is part of the Review Process File (RPF), which will be published alongside your paper.

4) A complete author checklist, which you can download from our author guidelines. Please insert information in the checklist that is also reflected in the manuscript. The completed author checklist will also be part of the RPF.

5) Please note that all corresponding authors are required to supply an ORCID ID for their name upon submission of a revised manuscript.

6) It is mandatory to include a 'Data Availability' section after the Materials and Methods. Before submitting your revision, primary datasets produced in this study need to be deposited in an appropriate public database, and the accession numbers and database listed under 'Data Availability'. Please remember to provide a reviewer password if the datasets are not yet public.

In case you have no data that requires deposition in a public database, please state so in this section. Note that the Data Availability Section is restricted to new primary data that are part of this study.

7) For data quantification: please specify the name of the statistical test used to generate error bars and P values, the number (n) of independent experiments (specify technical or biological replicates) underlying each data point and the test used to calculate p-values in each figure legend. The figure legends should contain a basic description of n, P and the test applied. Graphs must include a description of the bars and the error bars (s.d., s.e.m.).

8) At EMBO Press we ask authors to provide source data for the main manuscript figures. You will receive a separate email with instructions for providing source data with your revised manuscript, including how to upload and organize the files.

9) Our journal encourages inclusion of *data citations in the reference list* to directly cite datasets that were re-used and obtained from public databases. Data citations in the article text are distinct from normal bibliographical citations and should directly link to the database records from which the data can be accessed. In the main text, data citations are formatted as follows: "Data ref: Smith et al, 2001" or "Data ref: NCBI Sequence Read Archive PRJNA342805, 2017". In the Reference list,

data citations must be labeled with "[DATASET]". A data reference must provide the database name, accession number/identifiers and a resolvable link to the landing page from which the data can be accessed at the end of the reference.

10) We replaced Supplementary Information with Expanded View (EV) Figures and Tables that are collapsible/expandable online. A maximum of 5 EV Figures can be typeset. EV Figures should be cited as 'Figure EV1, Figure EV2" etc... in the text and their respective legends should be included in the main text after the legends of regular figures.

- For the figures that you do NOT wish to display as Expanded View figures, they should be bundled together with their legends in a single PDF file called *Appendix*, which should start with a short Table of Content. Appendix figures should be referred to in the main text as: "Appendix Figure S1, Appendix Figure S2" etc.

- Additional Tables/Datasets should be labeled and referred to as Table EV1, Dataset EV1, etc. Legends have to be provided in a separate tab in case of .xls files. Alternatively, the legend can be supplied as a separate text file (README) and zipped together with the Table/Dataset file.

11) The paper explained: EMBO Molecular Medicine articles are accompanied by a summary of the articles to emphasize the major findings in the paper and their medical implications for the non-specialist reader. Please provide a draft summary of your article highlighting
- the medical issue you are addressing,
- the results obtained and
- their clinical impact.

This may be edited to ensure that readers understand the significance and context of the research. Please refer to any of our published articles for an example.

12) Author contributions: You will be asked to provide CRediT (Contributor Role Taxonomy) terms in the submission system. These replace a narrative author contribution section in the manuscript.

13) A Conflict of Interest statement should be provided in the main text.

14) Every published paper includes a 'Synopsis' to further enhance discoverability. Synopses are displayed on the journal webpage and are freely accessible to all readers. They include a short stand first (maximum of 300 characters, including space) as well as 2-5 one-sentences bullet points that summarizes the paper. Please write the bullet points to summarize the key NEW findings. They should be designed to be complementary to the abstract - i.e. not repeat the same text. We encourage inclusion of key acronyms and quantitative information (maximum of 30 words / bullet point). Please use the passive voice. Please attach these in a separate file or send them by email, we will incorporate them accordingly.

Please also suggest a striking image or visual abstract to illustrate your article as a PNG file 550 px wide x 300-600 px high.

15) Include a Reagents and Tools Table as part of the Methods section, which can be downloaded from our author guidelines.

16) As part of the EMBO Publications transparent editorial process initiative (see our Editorial at http://embomolmed.embopress.org/content/2/9/329), EMBO Molecular Medicine will publish online a Review Process File (RPF) to accompany accepted manuscripts.
In the event of acceptance, this file will be published in conjunction with your paper and will include the anonymous referee reports, your point-by-point response and all pertinent correspondence relating to the manuscript. Let us know whether you agree with the publication of the RPF and as here, if you want to remove or not any figures from it prior to publication.
Please note that the Authors checklist will be published at the end of the RPF.

EMBO Molecular Medicine has a "scooping protection" policy, whereby similar findings that are published by others during review or revision are not a criterion for rejection. Should you decide to submit a revised version, I do ask that you get in touch after three months if you have not completed it, to update us on the status.
Molecular Medicine.

I look forward to receiving your revised manuscript.

Yours sincerely,

Lise Roth

Lise Roth, PhD
Senior Editor
EMBO Molecular Medicine

***** Reviewer's comments *****

Referee #1 (Comments on Novelty/Model System for Author):

Some figures could benefit from improved clarity. Result interpretation could also benefit from a clearer detection of dead cells. These points are discussed in the full review.

Referee #1 (Remarks for Author):

In this manuscript, Chernyavska et al explore the use of retargeted adenoviruses to deliver anti-EGFR and anti-EpCAM IgA antibodies to cancer cells (HCT-116, MDA-MB-468) as a therapeutic strategy to promote neutrophil and macrophage recognition and destruction of cancer cells. They used a microfluidic device to establish a multi-culture including cancer cells (transduced with the virus), fibroblasts, and neutrophils or macrophages. Their results elegantly demonstrated production of IgA antibodies only in the target cells (i.e., cancer cells) without affecting other cells like fibroblasts. Additionally, they used fluorescence microscopy to track extracellular DNA in the microfluidic device, which was used as a proxy for neutrophil-mediated antibody-dependent cell cytotoxicity (ADCC). However, the use of an extracellular staining as a proxy to quantify cell death may cause some important challenges with result interpretation. The images show an extracellular DNA pattern that suggest neutrophils are dying by netosis and releasing their DNA (which is suggested by the authors in the text and supplementary figures as well). Additionally, there is a strong DNA signal that seems to originate in between the pillars delimitating the central chamber (where there are no cancer cells or their density is very low). Thus, these observations raise important concerns about how the assay can truly distinguish between dead tumor cells vs. tumor cells surrounded by neutrophil extracellular DNA (supplementary figure 12 actually seems to show cancer cells are often alive but surrounded by DNA). This technique is central to the manuscript since it's used across many figures to drive the main conclusions of the study (i.e., IgA coating of tumor cells leads to neutrophil-mediated ADCC and tumor cell killing). Otherwise, the manuscript is well written, although the figures could use some extra details like legend bars (e.g., Fig. 6) or more detailed schematics depicting the experimental sequence (e.g. Fig. 4 and 6 could use multiple schematics describing the order and time of each reagent/cell added to the microdevice). This manuscript presents exciting data about the potential therapeutic role of retargeted viruses to leverage neutrophil and macrophage ADCC (and ADCP). However, I would recommend including some additional data with a more clear and informative staining that supports the central hypothesis of the study.

Major
Fig. 3 - Some of the images seem to show the pillars that delimit the central chamber, suggesting the infected cells were very close to the lateral channels where the virus was perfused. This raises some interesting questions about the capacity of the virus to penetrate in a solid tumors. Have the authors seen/analyzed virus infection across the cahmber width? Plotting some metric like percentage of tumor infected cells as a function of their location in the chamber could provide interesting information since virus penetration in solid tumors is an important question.
Fig. 5 - Same question about the inclusion of antibody alone controls. Showing data analyzing tumor cell death in the presence of the antibodies alone would strengthen the manuscript. Additionally, this reviewer failed to see the difference between some graphs shown in Fig.4 compared with Fig. 5.
Fig. 6 - The overlay images appear very pixelated, making any interpretation very challenging (this is probably caused during the compression process that some journals use, but having clear images is critical for a manuscript that relies on them so heavily). It almost seems like most of the DNA staining does not overlay with cancer cells. The pattern shown could be compatible with NETs given the "stripes" pattern (https://www.caymanchem.com/news/quantifying-nets?srsltid=AfmBOoqeKtNaCzq_MOvycIZpC8GE4lEjkRN6ex446FCygT81Nreg5mZF). Also, there seems to be an intense signal in the spaces between pillars, which is not explained. Other papers evaluating ADCC (even microfluidic devices) have used conventional stainings like propidium iodide or cellEvent apoptosis detection reagent (both available in Thermo) in combination with cell trackers to clearly discriminate dead and live tumor/immune cells.
Is it possible that neutrophils are undergoing netosis? The authors seem to suggest this possibility. So, using a DNA staining in a co-culture where extracellular DNA is floating (as a results of netosis) makes result interpretation very difficult.
Additionally, having a condition with cells transduced with an empty virus (i.e., no EGFR or SIRP delivered) could also help clarify if the virus is triggering (or some viral proteins) are triggering netosis.
The MDA-MB-468 cell density seems to be significantly higher in the conditions treated with AdV compared with no virus (NT).

"Initially, we confirmed that our purified IgA antibodies could effectively induce neutrophil-mediated killing of cancer cells in this model (Figure S10)". The figure should include images showing the killing.

Minor:

"Furthermore, dimeric IgA, which is the most common form in mucosal tissues, is even more effective than monomeric IgA in Fc and F(ab)-mediated tumor cell killing mechanisms, the latter referring to mechanisms involving, for instance, inhibition of ligand binding and receptor downmodulation." Could be explained in more detail.
Fig 2. - consider adding text to the "IgA" labels in b and c panels to specify that those cells are producing Anti-EGFR IgA (right now the labels only say "IgA"). In panel d: would it make sense to include a fluorescence image showing the inability of the EGFR/EpCAM-retargeted virus to infect EpCAM/EGFR negative cells? That would prove the virus is selectively infecting the targeted cells (although the authors use fibroblasts as a negative control which partially alleviates this concern).
Fig. 3 - Why does the schematic shown in panel f have two regions labelled (one in blue and one in red)?
Fig.3/4 - I would suggest including the images of the device and schematics in the first panels of Fig. 3 so the readers can rapidly understand the assay.
Fig. 4 - panels could benefit from more legends and text indicating what's shown in each panel/image. Controls showing antibody alone (i.e., no neutrophils) should be included to rule out cancer cell death due to the presence of the antibody (it's unlikely but it's an easy control that should be included).

Referee #2 (Comments on Novelty/Model System for Author):

It is an interesting work that has potential

Referee #2 (Remarks for Author):

In this study, Chernyavska et al. present a retargeted adenoviral approach that reprograms tumor cells to locally produce IgA antibodies together with a CD47-blocking SIRPα-Fc fusion protein, enabling effective targeting of antigens such as EGFR and EpCAM and promoting innate immune-mediated tumor killing. In FcαRI-transgenic, tumor-bearing mice, a single intratumoral injection induced robust IgA and SIRPα-Fc expression, increased immune-cell infiltration, and led to more than 50% tumor reduction. Dimeric IgA proved more potent than monomeric IgA, underscoring the value of localized production and multivalency in this setting. Overall, the experimental work is clear and compelling, but several points should be addressed to improve clarity and provide a more complete picture:

In Figure 1B, under reducing conditions for the heavy chain, the height/intensity of the bands does not appear to align well between the controls (lanes 2 and 4) and the supernatants (lanes 1 and 3). The authors should clarify this discrepancy and, if relevant, comment on possible technical or biological explanations.

In Figure 4E, there seems to be no discernible difference in neutrophil-mediated ADCC between the "anti-EGFR mIgA AdV" and "anti-EGFR dIgA AdV" groups. The authors are encouraged to comment on this finding in the Discussion and to explain how it fits with their overall model of enhanced efficacy for dimeric IgA.

In the MDA-MB-468 model, an anti-EGFR IgA control is not included. It would be important to clarify why this control is absent from the graph, especially given that an anti-EpCAM IgA control is included for the HCT116 model.

The authors should provide information on whether any safety-related issues were observed in the in vivo experiments. In particular, clarification on potential toxicological effects, adverse reactions, changes in body weight, or other indicators of treatment-related toxicity would help assess the translational relevance and safety profile of the proposed approach.

It would strengthen the manuscript if the Discussion were expanded to address the differences observed across the three in vivo models, including potential biological or technical reasons for these disparities. In addition, a more detailed analysis of the relationship between tumor growth dynamics and overall survival would further support the interpretation of the in vivo findings.

***** Reviewer's comments *****

Referee #1 (Comments on Novelty/Model System for Author):

Some figures could benefit from improved clarity. Result interpretation could also benefit from a clearer detection of dead cells. These points are discussed in the full review.

Referee #1 (Remarks for Author):

1. In this manuscript, Chernyavska et al explore the use of retargeted adenoviruses to deliver anti-EGFR and anti-EpCAM IgA antibodies to cancer cells (HCT-116, MDA-MB-468) as a therapeutic strategy to promote neutrophil and macrophage recognition and destruction of cancer cells. They used a microfluidic device to establish a multi-culture including cancer cells (transduced with the virus), fibroblasts, and neutrophils or macrophages. Their results elegantly demonstrated production of IgA antibodies only in the target cells (i.e., cancer cells) without affecting other cells like fibroblasts. Additionally, they used fluorescence microscopy to track extracellular DNA in the microfluidic device, which was used as a proxy for neutrophil-mediated antibody-dependent cell cytotoxicity (ADCC). However, the use of an extracellular staining as a proxy to quantify cell death may cause some important challenges with result interpretation.

Response: We appreciate the reviewer raising this important point regarding the interpretation of the cytotoxicity assays. We agree that the presence of extracellular DNA (e.g., NETs) in neutrophil co-cultures presents a challenge when using DNA-intercalating dyes like CellTox Green.
To ensure that we quantified actual cancer cell death rather than extracellular DNA, we employed a strict masking strategy during image analysis. We did not quantify total fluorescence. Instead, we created a binary mask of the cancer cells (identified by cytoplasmic CellTrace Red) and a mask of the dead material (CellTox Green). Cell death was strictly defined as the overlapping area between these two masks. Consequently, extracellular DNA (green signal) that did not colocalize with a cancer cell (red signal) was excluded from the quantification. We have clarified this quantification strategy in the Methods section (section 4.13) to prevent ambiguity.

Our addition in the manuscript text (page 31, lines 776-778):
"Crucially, any CellTox Green signal not co-localizing with the CellTrace Far Red mask (e.g., extracellular DNA from NETs or dead neutrophils) was excluded from the quantification."

2. The images show an extracellular DNA pattern that suggest neutrophils are dying by netosis and releasing their DNA (which is suggested by the authors in the text and supplementary figures as well). Additionally, there is a strong DNA signal that seems to originate in between the pillars delimitating the central chamber (where there are no cancer cells or their density is very low). Thus, these observations raise important concerns about how the assay can truly distinguish between dead tumor cells vs. tumor cells surrounded by neutrophil extracellular DNA (supplementary figure 12 actually seems to show cancer cells are often alive but surrounded by DNA). This technique is central to the manuscript since it's used across many figures to drive the main conclusions of the study (i.e., IgA coating of tumor cells leads to neutrophil-mediated ADCC and tumor cell killing).

Response: We acknowledge the challenges in getting highly precise estimates of cell death with our approach. As explained in our response to point 1, since we rely on measuring overlap, there is little to no risk that regions with strong DNA signals but without or little cancer cell signal (i.e., CellTrace Far Red) would yield significant bias. This was supported by the observation of dead neutrophils (and associated CellTox Green signal) in conditions without antibodies or regions with few tumor cells, where considerably less overlap was seen. We have now added a panel in Appendix Fig, S7 (panel c), with visual support of our step-wise masking approach for quantifying tumor cell death using several illustrative examples. With respect to wrapping of DNA (e.g. from NETs) around live cancer cells, at the resolution we used for analyzing confocal slices (HC PL FLUOTAR 10×/0.30 dry objective), there will only be a minimal overlap of area erroneously considered as dead cell area by our approach (see bottom row of panel c of Appendix Fig. S7 below, for an illustrative example). Besides adding this panel to Appendix Fig. S7, we now refer explicitly to this limitation of our methodology in our manuscript (see below). To further support our data, we would like to emphasize that our classical ^{51}Cr

release-based ADCC assays (Appendix Fig. S3) and our in vivo efficacy experiments (showing overall tumor reduction), which rely on completely different methodologies, yielded results consistent with our microscopy-based ADCC quantification approach on-chip.

Our addition in the manuscript text (page 18, lines 443-445):

"Our ADCC assays rely on quantification of overlap between tumor cells and a DNA stain indicating dead cells using a 10× objective, which has limited accuracy when dead neutrophils and tumor cells are very close by."

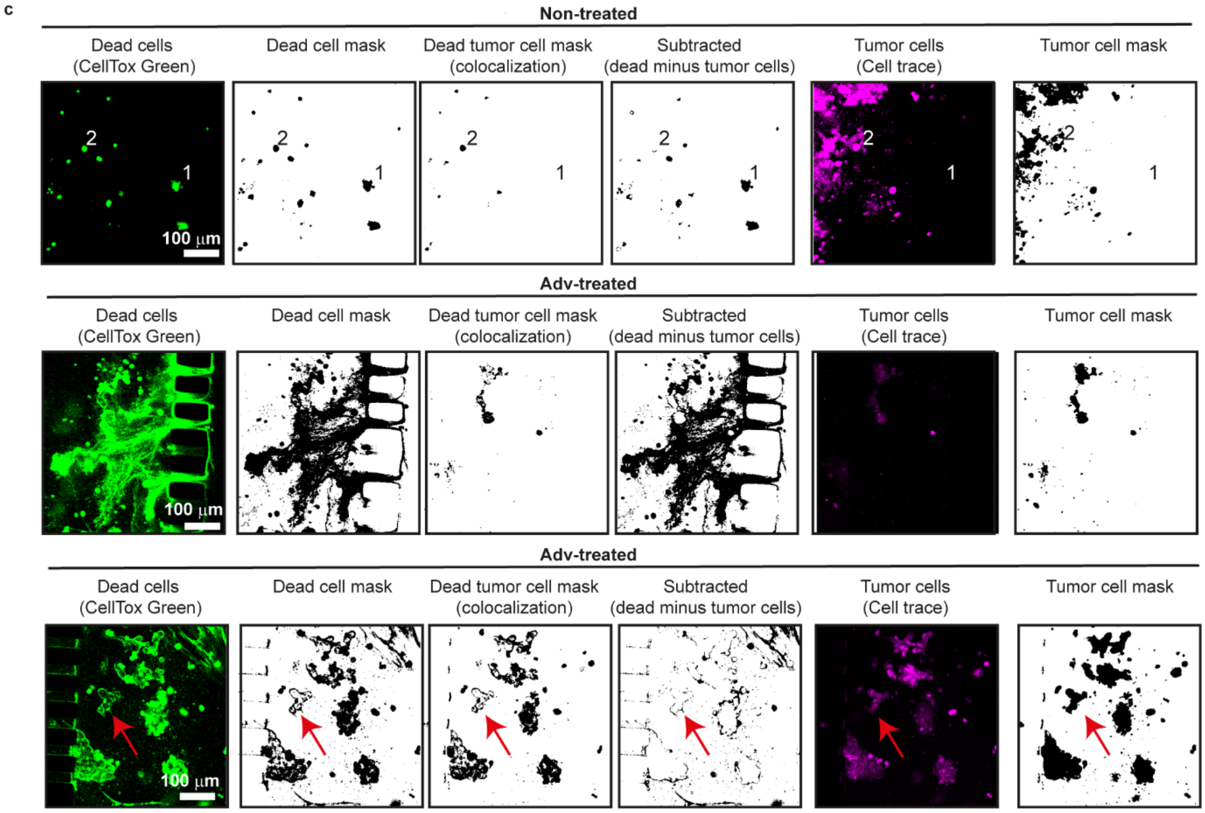

Appendix Figure S7c text:

(c) Top: Illustrative example of step-wise masking approach for non-treated HCT116 cells, which is part of the ADCC quantification approach. Dead cells are seen that do not show overlap with tumor cells (# 1: no overlap of 'Tumor cell mask' with 'Dead cell mask'), and which likely reflect dead neutrophils. Some dead tumor cells are still seen (# 2: 'Tumor cell mask overlaps with 'Dead cell mask'). Middle: Illustrative example of Anti EpCAM IgA AdV-treated HCT116 cells (targeted to EGFR), where a region is shown with high CellTox green signal but low tumor cell signal, indicating many dead neutrophils. Since only the colocalization counts toward tumor cell killing, the large amount of CellTox green signal does not negatively affect the quantification analyses, which are based on the colocalization signal. Bottom: Example of wrapping DNA around live tumor cells and associated analyses, for HCT116 cells treated with anti-EpCAM IgA-encoding AdV (targeted to EGFR). The red arrow illustrates an example where live tumor cells are wrapped with DNA (CellTox green staining). The dead cell mask clearly shows that only the outer rim is considered positive, but that the core area shows no overlap, indicating limited bias.

Otherwise, the manuscript is well written, although the figures could use some extra details like legend bars (e.g., Fig. 6) or more detailed schematics depicting the experimental sequence (e.g. Fig. 4 and 6 could use multiple schematics describing the order and time of each reagent/cell added to the microdevice).

Response: We interpret the reviewer's request for 'legend bars' as a request for scale bars in the microscopy images. While a scale bar was included in the first panel of the original Fig. 6a, we have now added scale bars

to the first image of each row in the revised Fig. 6 and other relevant figures (e.g., Fig. 2, 3, 4, 7) to ensure the magnification is immediately clear to the reader in every panel.
We additionally added schematics visually depicting the main order of steps in Fig. 4 and Fig. 6, to aid understanding of the sequence of events.

This manuscript presents exciting data about the potential therapeutic role of retargeted viruses to leverage neutrophil and macrophage ADCC (and ADCP). However, I would recommend including some additional data with a more clear and informative staining that supports the central hypothesis of the study.

Major
Fig. 3 - Some of the images seem to show the pillars that delimit the central chamber, suggesting the infected cells were very close to the lateral channels where the virus was perfused. This raises some interesting questions about the capacity of the virus to penetrate in a solid tumors. Have the authors seen/analyzed virus infection across the chamber width? Plotting some metric like percentage of tumor infected cells as a function of their location in the chamber could provide interesting information since virus penetration in solid tumors is an important question.

Response: We have done this extensive characterization in a previous study with Alexa Fluor-labeled EGFR-retargeted adenoviral particles and EGFR-retargeted fluorescent protein encoding adenoviral particles (iRFP670, so not encoding IgA). Please see Fig. 4C for the on-chip visualization of the trafficking of the retargeted adenovirus and supplementary Fig. 7 of Chernyavska et al. (https://doi.org/10.1038/s41598-019-54939-9) for a quantification of the distribution of Alexa fluor 488-labeled, EGFR-targeted adenoviral vector across the tumor-on-a-chip. Intensity profiles of the fluorescent signal distribution indicated that even though most particles stayed close to the entry point, viral vectors were still enriched at regions where tumor spheroids were present. This transport also resulted in effective transduction of tumor spheroids on chip away from the collagen matrix entry point, as shown by iRFP670 visualization in optically cleared tumors spheroids on-chip (Figure 4e of abovementioned paper). The current study builds upon those earlier results. Thus, combining our previous results with our current "biofactory approach", which relies on secreted antibodies, this mechanistically explains the full coverage of tumor cells throughout the chip that we consistently observe.

Fig. 5 - Same question about the inclusion of antibody alone controls. Showing data analyzing tumor cell death in the presence of the antibodies alone would strengthen the manuscript. Additionally, this reviewer failed to see the difference between some graphs shown in Fig.4 compared with Fig. 5.

Response: We agree that antibody alone controls are highly relevant. In the main manuscript, our focus has been on comparing the various adenoviral vector-based approaches. Nonetheless, we have included a variety of controls with only antibodies (so without viral vectors, also referred to as 'pure protein'), which were mostly reported in the supplements in our original submission. Here is the overview and their location in the present manuscript:

- 2D ADCC – Appendix Fig. S3: Validation of Fc/Fab function of the antibody construct in 2D
- On-chip ADCC – Appendix Fig. S7: Validation that neutrophils kill cancer cells on-chip with pure protein
- On-chip ADCC – Appendix Fig. S9: Benchmarking for A-431 skin cancer model (anti-EGFR IgA alone vs. combination with SIRPα-Fc was tested
- On-chip ADCC – Fig. 6: Specificity control (killing of tumor cells vs. fibroblasts).
- On-chip ADCP – Fig. 4g: Pure antibody was used as a positive control for macrophage phagocytosis.

Besides the above controls, we also performed a number of pure antibody conditions in a previous tumor-on-a-chip study using anti-EGFR IgA antibodies and a CD47 blocker (Chernyavska et al. Organs-on-chips: https://doi.org/10.1016/j.ooc.2022.100019). Since the current study builds upon this earlier work, we consider it appropriate to include antibody-only conditions mainly as controls in the Supplement (Appendix).
Regarding the distinction between the graphs in Fig. 4 and 5, Fig. 4 establishes the baseline efficacy of the viral vector delivering IgA as a monotherapy (specifically comparing monomeric versus dimeric formats). In contrast, Fig. 5 introduces the combination therapy, demonstrating how the co-delivery of the CD47-blocking

SIRPα-Fc payload synergizes with the locally produced IgA to significantly enhance tumor cell killing, with monotherapies included as references.

Fig. 6 - The overlay images appear very pixelated, making any interpretation very challenging (this is probably caused during the compression process that some journals use, but having clear images is critical for a manuscript that relies on them so heavily).
It almost seems like most of the DNA staining does not overlay with cancer cells. The pattern shown could be compatible with NETs given the "stripes" pattern (https://www.caymanchem.com/news/quantifying-nets?srsltid=AfmBOoqeKtNaCzq_MOvycIZpC8GE4lEjkRN6ex446FCygT81Nreg5mZF).

Response: We apologize for the poor image quality in the PDF; we will ensure that high-resolution images are provided in the final submission to facilitate interpretation.
Regarding the DNA staining pattern, the reviewer is absolutely correct. The 'striped' extracellular DNA signals observed indeed likely correspond to Neutrophil Extracellular Traps (NETs) or DNA released from dying neutrophils, which is why they do not overlay with the cancer cells.
However, this lack of overlay is exactly what ensures the specificity of our analyses. Our quantification algorithm does not measure total DNA fluorescence. Instead, it explicitly calculates cancer cell death based only on the colocalization of the DNA signal (CellTox Green) within the cytoplasm of the labeled cancer cells (CellTrace Red). Any DNA signal that does not overlay with a cancer cell (e.g., NETs or neutrophil DNA) is excluded from the quantification. This strict masking strategy allows us to distinguish actual cancer cell death from the background of neutrophil-derived DNA. Several examples of our workflow were given in our earlier response (**Fig. A-C**; see above) to visually illustrate how our masking approach ensures specificity.

Also, there seems to be an intense signal in the spaces between pillars, which is not explained.

Response: We appreciate the reviewer noting this detail. The intense signal observed in the spaces between the pillars (microtunnels) corresponds to extracellular DNA, likely originating from neutrophils. In our microfluidic model, neutrophils actively migrate through these narrow pillars to enter the tumor compartment, and this physical constriction, combined with the release of NETs, leads to the accumulation of DNA material at these structural interfaces.
We wish to reassure the reviewer that this signal is not an artifact that affects our data. As mentioned in our previous response above, our quantification algorithm strictly masks for the colocalization of the DNA signal within the cytoplasm of the cancer cells. Since the 'pillar' signal is extracellular and spatially distinct from the cancer cell bodies, it is automatically excluded from our cell death quantification. See **Fig. B** (above) for a concrete example of our analysis pipeline that supports this point.

We have added the following text on this in figure legend 6a (page 57, line 1397-1400):
"Please note that the green signal in the microtunnels likely corresponds to an accumulation of extracellular DNA from neutrophils, which is not taken into account in the analyses as they rely on quantification of overlap with stained tumor cells

And in the Methods section 4.13 (page 31, lines 776-778):
"Crucially, any CellTox Green signal not co-localizing with the CellTrace Far Red mask (e.g., extracellular DNA from NETs or dead neutrophils) was excluded from the quantification."

Other papers evaluating ADCC (even microfluidic devices) have used conventional stainings like propidium iodide or cellEvent apoptosis detection reagent (both available in Thermo) in combination with cell trackers to clearly discriminate dead and live tumor/immune cells.

Response: We appreciate the suggestion but we specifically selected CellTox Green for technical reasons suited to our long-term, real-time microfluidic assays. Unlike CellEvent, which is restricted to apoptosis detection, CellTox Green detects membrane compromise from any cell death mechanism, including necrosis and trogoptosis, ensuring that we do not underestimate neutrophil-mediated killing. Furthermore, CellTox Green is non-toxic and compatible with long (overnight) incubations, whereas propidium iodide can exhibit toxicity during prolonged exposure. Finally, since propidium iodide operates on the same DNA-intercalation principle as CellTox Green, it would stain extracellular DNA (NETs) in the exact same manner. For the above

reasons, we considered CellTox green as the preferred choice to clearly discriminate dead and live tumor/immune cells, when used together with a cancer cell cytoplasmic stain.

Is it possible that neutrophils are undergoing netosis? The authors seem to suggest this possibility. So, using a DNA staining in a co-culture where extracellular DNA is floating (as a results of netosis) makes result interpretation very difficult.

Response: We agree that NETs created by neutrophil NETosis generate significant amounts of extracellular DNA. However, our quantification overcomes this difficulty by strictly measuring colocalization: cell death is only registered when the DNA signal (CellTox Green) overlaps with the cancer cell mask (CellTrace Red). See our response to the previous comment and **Fig. B** (above) for a stepwise visual example of our analysis approach regarding this issue. Consequently, extracellular DNA—including the intense signals observed between micropillars as seen in **Fig. B**—is automatically excluded from the analyses, ensuring that no bias occurs.

Additionally, having a condition with cells transduced with an empty virus (i.e., no EGFR or SIRP delivered) could also help clarify if the virus is triggering (or some viral proteins) are triggering netosis.

Response: We addressed this concern by including a mock adenoviral vector control in our experiments, specifically a viral vector encoding an irrelevant fluorescent protein (iRFP) instead of the IgA-based therapeutic payloads or the SIRPα-Fc. As demonstrated in Fig. 4e, tumor cells transduced with this control virus did not elicit significantly different neutrophil-mediated cytotoxicity (ADCC) compared to the untreated background, and nothing resembling NETs was observed in this condition (See **Fig. A** below an example image of Adv-iRP-treated MDA-MB-468 cells, EpCAM-targeted). We only observed significant NET formation when there also was significant tumor cell killing, which is the reason why we hypothesize that NETosis may be a mechanism contributing to the cytotoxicity.

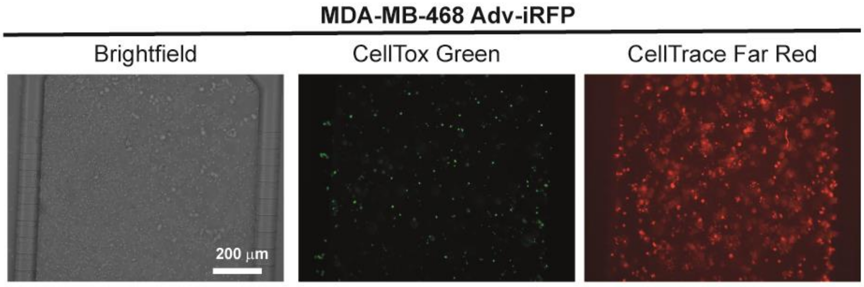

Figure A. Example of mock treated (Adv-iRP) MDA-MB-468 cells (EpCAM-targeted). Very little ADCC and no NET formation was observed in conditions without AdV encoding anti-EGFR or anti-EpCAM IgA. See Fig. 4e for quantification of ADCC results.

The MDA-MB-468 cell density seems to be significantly higher in the conditions treated with AdV compared with no virus (NT).

Response: We assume the reviewer is referring to Fig. 6b (originally Fig. 6a). We acknowledge the reviewer's observation regarding the apparent difference in cell density in the representative images for MDA-MD-468. Yet, all conditions were seeded at identical densities (same cell preparation) and cultured for the same duration. We inspected the other images, but there are always some apparent discrepancies in either the tumor cell compartment or fibroblast compartment. The visual discrepancy is hence attributable to normal biological variation in cell distribution and field-of-view selection. Importantly, our functional assays quantify cell death as a percentage of the total cancer cell area within each specific field of view, thereby normalizing for any local variations in cell density and ensuring the results remain robust despite this variation.

"Initially, we confirmed that our purified IgA antibodies could effectively induce neutrophil-mediated killing of cancer cells in this model (Figure S10)". The figure should include images showing the killing.

Response: We agree with the reviewer that visual evidence is important to substantiate the quantitative findings. To more clearly show our evidence, we have revised Fig. 6a, where we now include representative fluorescence microscopy images of protein controls, providing evidence that purified IgA antibodies can effectively induce neutrophil-mediated killing of cancer cells in our model, while simultaneously also addressing specificity (previously Supplementary Fig. S14). The added images correspond to the quantification in Fig. 6b and clearly visualize neutrophil-mediated killing by showing the accumulation of the cell death marker (CellTox Green) within the target cancer cells in the presence of purified IgA antibodies, providing direct visual confirmation of the ADCC activity.

Minor:

"Furthermore, dimeric IgA, which is the most common form in mucosal tissues, is even more effective than monomeric IgA in Fc and F(ab)-mediated tumor cell killing mechanisms, the latter referring to mechanisms involving, for instance, inhibition of ligand binding and receptor downmodulation." Could be explained in more detail.

Response: We thank the reviewer for this suggestion. We have adjusted the text in the Introduction to explicitly describe the mechanistic basis for the superior efficacy of dimeric IgA. We now clarify that this enhanced potency is driven by the increased avidity of the tetravalent dimer, which leads to superior receptor blockade/cross-linking (F(ab)-mediated effects) and more potent clustering of FcαRI on effector cells (Fc-mediated effects).

The modified text is (page 3-4, lines 65-70):
"Furthermore, engineered dimeric IgA is even more effective than monomeric IgA in Fc and F(ab)-mediated tumor cell killing mechanisms.22 This superior efficacy is largely attributed to the tetravalent nature of dimeric IgA (vs. bivalent monomers), which provides increased avidity. This enhances F(ab)-mediated effects such as the inhibition of ligand binding and receptor downmodulation through increased receptor cross-linking, while simultaneously promoting stronger clustering of FcαRI on myeloid effector cells to boost Fc-mediated cytotoxicity."

Fig 2. - consider adding text to the "IgA" labels in b and c panels to specify that those cells are producing Anti-EGFR IgA (right now the labels only say "IgA"). In panel d: would it make sense to include a fluorescence image showing the inability of the EGFR/EpCAM-retargeted virus to infect EpCAM/EGFR negative cells? That would prove the virus is selectively infecting the targeted cells (although the authors use fibroblasts as a negative control which partially alleviates this concern).

Response: We thank the reviewer for these suggestions to improve clarity.

1. Labeling: We have updated the labels in Fig. 2b and 2c to explicitly read 'Anti-EpCAM IgA' and 'Anti-EGFR IgA' as requested.

2. Specificity Control: Regarding the request for a negative control image in (in panel 2d), we agree that validation of specificity is of key importance. However, as alluded to before, we demonstrate this already in a number of different experiments in the paper (e.g. Fig. 3c for binding specificity and Fig. 6 for specificity of functional targeting). Since we look at an exported protein, a specificity control for *infection specificity* would require a different encoded gene, e.g. a fluorescent protein that does not get exported. We have extensively performed such controls in previous studies for our viral vectors and extensively characterized and validated the DARPin-based retargeting platform (doi: 10.1038/s41598-019-54939-9; doi: 10.1038/s41467-017-02707-6; 10.1073/pnas.1213653110).

Fig. 3 - Why does the schematic shown in panel f have two regions labelled (one in blue and one in red)?

Response: We added the follow explanatory information in the figure legend of Fig. 3a (previously 3f) (page 53, lines 1306-1307):
"The approximate areas of the chip of the respective microscopy images are shown (blue: c-e; red: f,g)."

Fig.3/4 - I would suggest including the images of the device and schematics in the first panels of Fig. 3 so the readers can rapidly understand the assay.

Response: We thank the reviewer and have added visual support in Fig. 3 and 4. The design, experimental workflow and photo of the chip have now been introduced in Fig. 3a and 3b, to facilitate a timely understanding by the reader. Likewise, a schematic image of the protocols of the ADCC and ADCP experiments has been added in Fig. 4a.

Fig. 4 - panels could benefit from more legends and text indicating what's shown in each panel/image. Controls showing antibody alone (i.e., no neutrophils) should be included to rule out cancer cell death due to the presence of the antibody (it's unlikely but it's an easy control that should be included).

Response: We thank the reviewer for this suggestion. For the legends, we have updated these for Fig. 4 to clearly indicate the content of each panel and the timeline of the assay. With respect to the controls, we agree that ruling out direct antibody toxicity is essential. We would like to clarify that this control is intrinsic to our on-chip experimental design. In our protocol, tumor cells are transduced and produce IgA antibodies for 3 days prior to the addition of neutrophils.

We image these chips immediately before adding neutrophils (t=0) to establish a baseline. At this timepoint—after 72 hours of exposure to the locally produced antibody—we consistently observed negligible cell death (comparable to non-transduced controls), confirming that the antibody alone is not cytotoxic (see example image **Fig. B** below for AdV-produced anti-EpCAM IgA in HCT-116 cells). As a quantitative example to support this representative image, please also see below an image of numbers derived from image processing in **Fig. C**, with and without background correction, for a representative set of conditions, which indicate negligible cell death before adding neutrophils. Source data for the experiments will be provided, as required for publication in EMBO Mol Med.

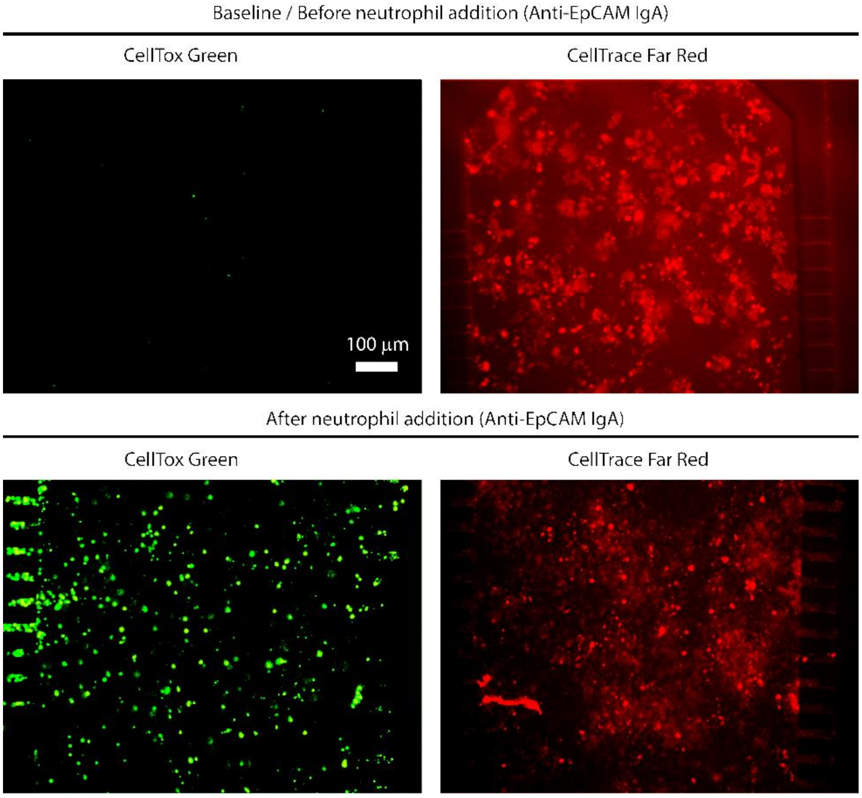

Figure B. Illustration of cell viability before and after adding neutrophils in HCT-116 cells treated with anti-EpCAM IgA-encoding adenoviruses. CellTox Green stains dead cells, CellTrace Far Red stains the cytoplasm of tumor cells.

			Before PMN			After PMN					
			Area value	% area	Normalized to red area	Area value	% area	Normalized to red area	Single normalized after-before	Average after	After-before
1		red area	243872	19,85	10	330157	26,87	249	240	250	240
	1	CellTox Green overlap	2365	0,19		82333	6,7				
		red area				393265	32	250	241		
	2	CellTox Green overlap				98492	8,02				
2		red area	94506	7,69	10	117134	9,53	239	229	285	275
	1	CellTox Green overlap	1015	0,08		28013	2,28				
		red area				119908	9,76	331	321		
	2	CellTox Green overlap				39680	3,23				
3		red area	58074	4,73	32	70565	5,74	319	319	358	326
	1	CellTox Green overlap	1822	0,15		22532	1,83				
		red area				80138	6,52	397	365		
	2	CellTox Green overlap				31798	2,59				
4		red area	135583	11,03	23	253664	20,64	151	151	219	196
	1	CellTox Green overlap	3081	0,25		38307	3,12				
		red area				299225	24,35	287	264		
	2	CellTox Green overlap				85729	6,98				
5		red area	90935	7,4	28	142445	11,59	354	326	341	312
	1	CellTox Green overlap	2566	0,21		50467	4,11				
		red area				99017	8,06	327	299		
	2	CellTox Green overlap				32360	2,63				
6		red area	93721	7,63	30	87841	7,15	315	285	306	276
	1	CellTox Green overlap	2786	0,23		27679	2,25				
		red area				103670	8,44	297	267		
	2	CellTox Green overlap				30786	2,51				

Figure C. Example data of raw processing of various conditions where anti-EpCAM IgA was expressed upon adenoviral transduction. The low values before addition of neutrophils (before PMN), as compared to values after addition of neutrophils (important numbers marked in yellow) indicate very low baseline cytotoxicity of expressed antibodies. 1,2: AdV EpCAM IgA MOI 50, retargeted to EpCAM. 3,4: AdV EpCAM IgA MOI 50 + AdV SIRPα-Fc MOI 5, both retargeted to EpCAM. 5,6: AdV EpCAM IgA MOI 50, retargeted to EGFR.

We have added a statement to the Fig. 4 legend explicitly clarifying that the baseline (t=0) measurement confirms the absence of direct antibody-mediated toxicity (page 54, lines 1334-1336):
"The baseline measurement at t=0, reflecting direct antibody-mediated toxicity, was typically low and subtracted from each condition to reach the depicted relative cell death."

Referee #2 (Comments on Novelty/Model System for Author):

It is an interesting work that has potential

Referee #2 (Remarks for Author):

In this study, Chernyavska et al. present a retargeted adenoviral approach that reprograms tumor cells to locally produce IgA antibodies together with a CD47-blocking SIRPα-Fc fusion protein, enabling effective targeting of antigens such as EGFR and EpCAM and promoting innate immune-mediated tumor killing. In FcαRI-transgenic, tumor-bearing mice, a single intratumoral injection induced robust IgA and SIRPα-Fc expression, increased immune-cell infiltration, and led to more than 50% tumor reduction. Dimeric IgA proved more potent than monomeric IgA, underscoring the value of localized production and multivalency in this setting. Overall, the experimental work is clear and compelling, but several points should be addressed to improve clarity and provide a more complete picture:

In Figure 1B, under reducing conditions for the heavy chain, the height/intensity of the bands does not appear to align well between the controls (lanes 2 and 4) and the supernatants (lanes 1 and 3). The authors should clarify this discrepancy and, if relevant, comment on possible technical or biological explanations.

Response: We thank the reviewer for this close inspection of the Western Blot data.
The differences in intensity of the bands are due to experimental design. We aimed to load the same amount of protein, but since we are comparing supernatants (conditioned media from transfection) with purified proteins, the loaded amounts also reflected production yield and are not fully normalized against the purified recombinant IgA antibodies. Regarding the height (migration) alignment, we acknowledge the slight shift in migration. As noted in our Discussion, the apparent molecular weight of IgA heavy chains can vary due to differential glycosylation patterns between the transiently produced supernatant samples and the purified benchmark controls, which in our case is the most likely reason for the difference. Additionally, supernatant

samples contain serum proteins from the cell culture media, which can impact the electrophoretic mobility as well. We added a comment in the Discussion to acknowledge this possibility as well.

The revised text is (page 15, lines 360-363):
"Interestingly, we observed a slightly higher molecular weight for the IgA heavy chains produced from our system, which might be attributed to differences in glycosylation patterns due to different production systems71, though we cannot exclude an impact of the loading matrix (i.e. supernatants contain many serum proteins that can impact electrophoretic mobility).

In Figure 4E, there seems to be no discernible difference in neutrophil-mediated ADCC between the "anti-EGFR mIgA AdV" and "anti-EGFR dIgA AdV" groups. The authors are encouraged to comment on this finding in the Discussion and to explain how it fits with their overall model of enhanced efficacy for dimeric IgA.

Response: We thank the reviewer for this observation. We agree that in the on-chip ADCC assay (Fig. 4e), the activities of the monomeric and dimeric anti-EGFR IgA conditions (as well as monomeric and dimeric anti-EpCAM IgA) were very similar, possibly because the high local concentration of antibodies in the confined microfluidic device saturated the neutrophil response. We added the following sentence in the results section (page 9, line 218):
"Hence, for both targets, monomeric and dimeric IgA performed similarly in this assay".

In the Discussion we added the following sentence (page 16, lines 392-396):
"We did not observe as clear superior activity of dimeric IgA compared to monomeric IgA in ADCC tumor-on-a-chip assays (e.g. in Fig. 4e, Fig. 5a,b) as compared to in vivo (Fig. 8), even though trends were consistently towards higher activity for dimeric IgA in both on-chip and in vivo assays. Possibly, saturation of the neutrophil ADCC response due to high local antibody production at the tested MOI reduced the size of the effect difference on-chip."

These observations fit with the overall superior efficacy of the dimeric IgA, which was clearly demonstrated in the in vivo therapeutic setting.

In the MDA-MB-468 model, an anti-EGFR IgA control is not included. It would be important to clarify why this control is absent from the graph, especially given that an anti-EpCAM IgA control is included for the HCT116 model.

Response: We assume the reviewer is referring to Fig. 4g, where there is an anti-EpCAM IgA control, but not an anti-EGFR IgA control. The rationale to omit the anti-EGFR IgA is both scientific and practical. Practically, we needed a positive control for the assay with each blood (effector cell) donation. Yet, we also obtained limited amounts of neutrophils from each blood donation, meaning we were limited in the number of conditions we could test. Scientifically, for Fig. 6b,c and Appendix Figs. S3, S7 and S9, we already included the anti-EGFR IgA control for the MDA-MD-468 model, which shows comparable activity to the viral vector-delivered IgA gene. The specific control condition in question for Fig. 4g was previously published by us (https://doi.org/10.1016/j.ooc.2022.100019). Similar results were seen as compared to the anti-EpCAM IgA control, which we now report for the first time. Since the results of this control were previously published and that there were limitations in how many conditions we could test, this additional anti-EGFR IgA control was not prioritized by us for the ADCP on-chip assay.

The authors should provide information on whether any safety-related issues were observed in the in vivo experiments. In particular, clarification on potential toxicological effects, adverse reactions, changes in body weight, or other indicators of treatment-related toxicity would help assess the translational relevance and safety profile of the proposed approach.

Response: We thank the reviewer for this important question regarding the safety profile. We closely monitored animal welfare throughout the in vivo experiments, and we are pleased to report that we did not observe any overt signs of treatment-related toxicity. In both the delivery study (Fig. 7) and the efficacy study (Fig. 8), no mouse lost more than 10% of its weight. In the delivery study, one mouse had transient weight

loss (<10%) due to an ear inflammation due to ear marking, which later resolved. In the efficacy study, several skin ulcers were seen across treatment conditions and controls, with no clear trend. Hence, ulcer formation is likely due to subcutaneous administration of tumor cells. (MDA-MB-468: 2 mice, 1× IgA monomer, 1× IgA monomer + CD47; B16F10, 2× IgA monomer + CD47; HCT116: 2× vehicle control, 3× IgA dimer) No signs of treatment-related toxicity or adverse behavioral changes were observed in any of the mice. These findings support the translational premise of our approach: that local "biofactories" can deliver potent immunotherapy without the systemic adverse effects (e.g., anemia from CD47 blockade) typically associated with these agents, as proteins are produced locally within the tumor and remain at very low or undetectable levels in the systemic circulation.

We now report on this more extensively in the manuscript:
Methods section (page 34, lines 860-862):
"Animal health, behavior, and body weights were monitored routinely throughout all in vivo studies to assess potential tumor model or treatment-related toxicity."

Results section (page 14, lines 344-348):
"Overall, the localized therapy approach demonstrated a favorable safety profile with no overt signs of systemic toxicity; no treatment-related behavioral changes or body weight losses exceeding 10% were observed. While transient subcutaneous ulcers occurred in a small subset of mice, these were distributed across both control and treatment groups, indicating they were most likely a consequence of tumor implantation rather than the viral therapy."

Discussion section (page 20, lines 494-497):
"Crucially, the localized production of these immunotherapeutic molecules within the tumor microenvironment appeared to circumvent systemic toxicities frequently observed by systemic administration, such as CD47-mediated anemia, reinforcing the translational safety and viability of this targeted viral vector platform."

It would strengthen the manuscript if the Discussion were expanded to address the differences observed across the three in vivo models, including potential biological or technical reasons for these disparities. In addition, a more detailed analysis of the relationship between tumor growth dynamics and overall survival would further support the interpretation of the in vivo findings.

We thank the reviewer for this suggestion. We have expanded the Discussion to briefly contextualize the inherent biological differences across the three models, noting factors such as the distinct immune environments (immunodeficient xenografts vs. the fully immunocompetent B16F10 syngeneic model) and varying target antigen densities.
However, regarding the request for a more detailed analysis of tumor growth dynamics versus overall survival, we must clarify that our primary rationale for utilizing three distinct in vivo models was to broadly validate the robustness and translational potential of our approach across diverse settings, rather than to directly compare the models to one another. Because these were designed as proof-of-concept efficacy studies, they were not statistically powered to conduct correlative analyses between growth kinetics and survival outcomes across the different tumor types. The requested analyses would therefore be underpowered. Appreciating the Reviewer's point, their value though, we have added a brief note to the Discussion clarifying this intended scope.

Added text in discussion (page 19, lines 466-469):
"We utilized three distinct in vivo models primarily to broadly validate the translational robustness of our platform across different immune environments (immunodeficient xenografts vs. the fully immunocompetent B16F10 syngeneic model) and target antigen densities."

29th Apr 2026

Dear Dr. Verdurmen,

Thank you for the submission of your revised manuscript to EMBO Molecular Medicine. The initial referees have evaluated your revised manuscript and as you will see below, they are satisfied with the revisions. We will therefore be able to accept your manuscript once the following editorial matters are addressed:

1/ Manuscript text:
- Please remove the highlighted text and only keep in track changes mode any new modification.
- Please spell out the authors' full names as they should be published on the manuscript title page.
- Update the headings and order of the sections in the manuscript text to: Abstract / The Paper Explained / Introduction / Results / Discussion / Methods / Data Availability / Acknowledgements / Disclosure and Competing Interests Statement / References / Figure Legends / Expanded View Figure Legends
- Please remove 'data not shown' (p. 14): as per our guidelines, all new data mentioned in the manuscript should be presented in the main or EV figures.
- Methods:
o Cells: please indicate whether the cells were authenticated.
o Human samples: If collected and within the bounds of privacy constraints report on age, sex and gender or ethnicity for all study participants (and update the checklist accordingly if applicable).
o Statistics: please include a statement on sample size and inclusion/exclusion criteria.
- Data availability: please remove "In alignment with FAIR principles, all data supporting the findings of this study are provided within the article and its supplementary information files" and "New materials are available upon reasonable request."
- Please remove Author contributions from the manuscript text, and provide instead CRediT (Contributor Role Taxonomy) terms in the submission system. These replace a narrative author contribution section in the manuscript.

2/ Figures:
- Please provide individual data points where possible.
- When n=2, please remove error bars and statistics.
- Please note that we can accommodate more than 5 EV figures, and we encourage you to make some of your appendix figures into EV figures.
- Please add callouts in the text for the EV figure panels.
- Figure 1B: please clarify if the same membrane was used for the top and bottom blots.
- Note that figure reused is allowed, but must be indicated in the figure legend (i.e. Fig. 2C and Appendix Fig. S4B; Fig. EV3A and Appendix Fig. S5A (exact panel should be mentioned); Fig. EV4A and Appendix Fig. S7C).
- Appendix: please add page numbers to the table of content. The resolution of the Appendix file is too low for microscopy images. This reduction in resolution is commonly caused by converting original 16-bit TIFF files to RGB format for publication. While this is not inherently problematic, it can raise concerns about image integrity for critical readers.
To avoid any misunderstanding and to meet EMBO Press standards, we kindly ask that you resubmit the Appendix file at the captured original data resolution. Please note that referee #1 also mentioned image quality.
- Please address the queries from our data editors in the figure legends:
1. Please note that the exact p values are not provided in the legends of figures 8A, E
2. Please note that information related to n is missing in the legends of figures 8A, C, E

3/ Synopsis:
I introduced minor edits in your synopsis, please let me know if you agree:
Retargeted adenoviral vectors transform tumor cells into biofactories secreting mono- or dimeric IgA and CD47 blockers. This strategy effectively engages neutrophils and macrophages for tumor destruction while minimizing the likelihood of systemic toxicity.
• Functional mono- and dimeric IgA are produced from single adenoviral vectors, enabling simultaneous secretion of heavy, light, and, for dimeric IgA, joining chains by transduced tumor cells.
• Neutrophil- and macrophage-mediated tumor cell killing is demonstrated in physiological 3D tumor-on-chip models, confirming the potent efficacy of locally produced IgA and SIRPa-Fc.
• Tumor growth inhibition (>50%) is achieved in xenograft models following a single intratumoral injection of the retargeted adenoviral vectors.
• Effective antibody concentrations are reached within the tumor microenvironment with negligible systemic exposure, thereby minimizing the likelihood of systemic toxicity.
The visual abstract should be resized to 550 x 300 pixels, and the font size should be increased to remain legible at these dimensions.

We note that you agree with the publication of the Review Process File.

I look forward to receiving your revised manuscript.

Yours sincerely,

Lise Roth

Lise Roth, PhD
Senior Editor
EMBO Molecular Medicine

***** Reviewer's comments *****

Referee #1 (Comments on Novelty/Model System for Author):

The manuscript still has some minor issues regarding image quality hat make data interpretation a bit challenging, this seems to be caused during the pdf conversion process, but the authors stated they will ensure image quality is preserved in the final version. This is probably addressable during the proffreading phase.

Referee #2 (Comments on Novelty/Model System for Author):

The combination of the tumor-on-a-chip platform with complementary in vitro assays and several in vivo models is appropriate and sufficient to support the main conclusions. No major limitations of the current model systems or ethical concerns are evident at this stage.

Referee #2 (Remarks for Author):

The authors have satisfactorily addressed the main concerns raised in the previous review round, especially regarding image analysis, controls and figure clarity. I am satisfied with the current version

The authors addressed the remaining editorial issues.

16th Jun 2026

Dear Dr. Verdurmen,

Thank you for submitting your revised files. I am pleased to inform you that your manuscript is accepted for publication and is now being sent to our publisher to be included in the next available issue of EMBO Molecular Medicine.

Your manuscript will be processed for publication by EMBO Press. It will be copy edited and you will receive page proofs prior to publication. Please note that you will be contacted by Springer Nature Author Services to complete licensing and payment information.

You may qualify for financial assistance for your publication charges - either via a Springer Nature fully open access agreement or an EMBO initiative. Check your eligibility: https://link.springer.com/journal/44321/how-to-publish-with-us

Should you be planning a Press Release on your article, please notify the production team via this form in order to coordinate publication and release dates: https://forms.cloud.microsoft/e/eH4A8JVP6g

If you have any questions, please do not hesitate to contact the Editorial Office.

Thank you for your contribution to EMBO Molecular Medicine!

With kind regards,

Lise Roth

Lise Roth, Ph.D
Senior Editor
EMBO Molecular Medicine

>>> Please note that it is EMBO Molecular Medicine policy for the transcript of the editorial process (containing referee reports and your response letter) to be published as an online supplement to each paper. If you do NOT want this, you will need to inform the Editorial Office via email immediately. More information is available here: https://link.springer.com/partners/embo-press/editorial-policies#Peer%20review

EMBO Press Author Checklist

Corresponding Author Name: Dr. Wouter P.R. Verdurmen	
Journal Submitted to: EMBO Molecular Medicine	
Manuscript Number: EMM-2025-22997	

USEFUL LINKS FOR COMPLETING THIS FORM
The EMBO Journal - Author Guidelines
EMBO Reports - Author Guidelines
Molecular Systems Biology - Author Guidelines
EMBO Molecular Medicine - Author Guidelines

Reporting Checklist for Life Science Articles (updated January
This checklist is adapted from Materials Design Analysis Reporting (MDAR) Checklist for Authors. MDAR establishes a minimum set of requirements in transparent reporting in the life sciences (see Statement of Task: 10.31222/osf.io/9sm4x). Please follow the journal's guidelines in preparing your
Please note that a copy of this checklist will be published alongside your article.

Abridged guidelines for figures
1. Data
The data shown in figures should satisfy the following conditions:
- the data were obtained and processed according to the field's best practice and are presented to reflect the results of the experiments in an accurate and unbiased manner.
- ideally, figure panels should include only measurements that are directly comparable to each other and obtained with the same assay.
- plots include clearly labeled error bars for independent experiments and sample sizes. Unless justified, error bars should not be shown for technical
- if n<5, the individual data points from each experiment should be plotted. Any statistical test employed should be justified.
- Source Data should be included to report the data underlying figures according to the guidelines set out in the authorship guidelines on Data

2. Captions
Each figure caption should contain the following information, for each panel where they are relevant:
- a specification of the experimental system investigated (eg cell line, species name).
- the assay(s) and method(s) used to carry out the reported observations and measurements.
- an explicit mention of the biological and chemical entity(ies) that are being measured.
- an explicit mention of the biological and chemical entity(ies) that are altered/varied/perturbed in a controlled manner.
- the exact sample size (n) for each experimental group/condition, given as a number, not a range;
- a description of the sample collection allowing the reader to understand whether the samples represent technical or biological replicates (including how many animals, litters, cultures, etc.).
- a statement of how many times the experiment shown was independently replicated in the laboratory.
- definitions of statistical methods and measures:
  - common tests, such as t-test (please specify whether paired vs. unpaired), simple $\chi 2$ tests, Wilcoxon and Mann-Whitney tests, can be unambiguously identified by name only, but more complex techniques should be described in the methods section;
  - are tests one-sided or two-sided?
  - are there adjustments for multiple comparisons?
  - exact statistical test results, e.g., P values = x but not P values < x;
  - definition of 'center values' as median or average;
  - definition of error bars as s.d. or s.e.m.

Please complete ALL of the questions below.
Select "Not Applicable" only when the requested information is not relevant for your study.

Materials

Newly Created Materials	Information included in the manuscript?	In which section is the information available? (Reagents and Tools Table, Materials and Methods, Figures, Data Availability Section)
New materials and reagents need to be available; do any restrictions apply?	Yes	Data availability section

Antibodies	Information included in the manuscript?	In which section is the information available? (Reagents and Tools Table, Materials and Methods, Figures, Data Availability Section)
For **antibodies** provide the following information:<br>- Commercial antibodies: RRID (if possible) or supplier name, catalogue number and or/clone number<br>- Non-commercial: RRID or citation	Yes	Reagents and Tools Table

DNA and RNA sequences	Information included in the manuscript?	In which section is the information available? (Reagents and Tools Table, Materials and Methods, Figures, Data Availability Section)
**Short novel DNA or RNA including primers, probes**: provide the sequences.	Yes	Methods

Cell materials	Information included in the manuscript?	In which section is the information available? (Reagents and Tools Table, Materials and Methods, Figures, Data Availability Section)
**Cell lines:** Provide species information, strain. Provide accession number in repository **OR** supplier name, catalog number, clone number, and/**OR** RRID.	Yes	Reagent and Tools Table
**Primary cultures:** Provide species, strain, sex of origin, genetic modification status.	Yes	Methods
Report if the cell lines were recently **authenticated** (e.g., by STR profiling) and tested for mycoplasma contamination.	Yes	Methods

Experimental animals	Information included in the manuscript?	In which section is the information available? (Reagents and Tools Table, Materials and Methods, Figures, Data Availability Section)
**Laboratory animals or Model organisms:** Provide species, strain, sex, age, genetic modification status. Provide accession number in repository **OR** supplier name, catalog number, clone number, **OR** RRID.	Yes	Reagents and Tools Table
**Animal observed in or captured from the field:** Provide species, sex, and age where possible.	Not Applicable	
Please detail **housing and husbandry conditions**.	Yes	Methods

Plants and microbes	Information included in the manuscript?	In which section is the information available? (Reagents and Tools Table, Materials and Methods, Figures, Data Availability Section)
**Plants:** provide species and strain, ecotype and cultivar where relevant, unique accession number if available, and source (including location for collected wild specimens).	Not Applicable	
**Microbes:** provide species and strain, unique accession number if available, and source.	Not Applicable	

Human research participants	Information included in the manuscript?	In which section is the information available? (Reagents and Tools Table, Materials and Methods, Figures, Data Availability Section)
If collected and within the bounds of privacy constraints report on age, sex and gender or ethnicity for all study participants.	Not Applicable	

Core facilities	Information included in the manuscript?	In which section is the information available? (Reagents and Tools Table, Materials and Methods, Figures, Data Availability Section)
If your work benefited from core facilities, was their service mentioned in the acknowledgments section?	Yes	Acknowledgements

Design

Study protocol	Information included in the manuscript?	In which section is the information available? (Reagents and Tools Table, Materials and Methods, Figures, Data Availability Section)
If study protocol has been **pre-registered, provide DOI in the manuscript**. For clinical trials, provide the trial registration number **OR** cite DOI.	Not Applicable	
Report the **clinical trial registration number** (at ClinicalTrials.gov or equivalent), where applicable.	Not Applicable	

Laboratory protocol	Information included in the manuscript?	In which section is the information available? (Reagents and Tools Table, Materials and Methods, Figures, Data Availability Section)
Provide DOI OR other citation details if **external detailed step-by-step protocols** are available.	Not Applicable	

Experimental study design and statistics	Information included in the manuscript?	In which section is the information available? (Reagents and Tools Table, Materials and Methods, Figures, Data Availability Section)
Include a statement about **sample size** estimate even if no statistical methods were used.	Not Applicable	
Were any steps taken to minimize the effects of subjective bias when allocating animals/samples to treatment (e.g. **randomization procedure**)? If yes, have they been described?	Yes	Methods
Include a statement about **blinding** even if no blinding was done.	Yes	Methods
Describe **inclusion/exclusion criteria** if samples or animals were excluded from the analysis. Were the criteria pre-established?<br><br>If sample or data points were omitted from analysis, report if this was due to attrition or intentional exclusion and provide justification.	Yes	Methods
For every figure, are **statistical tests** justified as appropriate? Do the data meet the assumptions of the tests (e.g., normal distribution)? Describe any methods used to assess it. Is there an estimate of variation within each group of data? Is the variance similar between the groups that are being statistically compared?	Yes	Methods

Sample definition and in-laboratory replication	Information included in the manuscript?	In which section is the information available? (Reagents and Tools Table, Materials and Methods, Figures, Data Availability Section)
In the figure legends: state number of times the experiment was **replicated** in laboratory.	Yes	Figures
In the figure legends: define whether data describe **technical or biological replicates**.	Yes	Figures

Ethics

Ethics	Information included in the manuscript?	In which section is the information available? (Reagents and Tools Table, Materials and Methods, Figures, Data Availability Section)
Studies involving **human participants**: State details of **authority granting ethics approval** (IRB or equivalent committee(s), provide reference number for approval.	Yes	Methods
Studies involving **human participants**: Include a statement confirming that **informed consent** was obtained from all subjects and that the experiments conformed to the principles set out in the WMA Declaration of Helsinki and the Department of Health and Human Services Belmont Report.	Yes	Methods
Studies involving **human participants: For** publication of **patient photos**, include a statement confirming that consent to publish was obtained.	Not Applicable	
Studies involving experimental **animals**: State details of **authority granting ethics approval** (IRB or equivalent committee(s), provide reference number for approval. Include a statement of compliance with ethical regulations.	Yes	Methods
Studies involving **specimen and field samples:** State if relevant **permits** obtained, provide details of authority approving study; if none were required, explain why.	Not Applicable	

Dual Use Research of Concern (DURC)	Information included in the manuscript?	In which section is the information available? (Reagents and Tools Table, Materials and Methods, Figures, Data Availability Section)
Could your study fall under dual use research restrictions? Please check biosecurity documents and list of **select agents and toxins** (CDC): https://www.selectagents.gov/sat/list.htm	Not Applicable	
If you used a select agent, is the security level of the lab appropriate and reported in the manuscript?	Not Applicable	
If a study is subject to dual use research of concern regulations, is the name of the **authority granting approval and reference number** for the regulatory approval provided in the manuscript?	Not Applicable	

Reporting

The MDAR framework recommends adoption of discipline-specific guidelines, established and endorsed through community initiatives. Journals have their own policy about requiring specific guidelines and recommendations to complement MDAR.

Adherence to community standards	Information included in the manuscript?	In which section is the information available? (Reagents and Tools Table, Materials and Methods, Figures, Data Availability Section)
State if relevant guidelines or checklists (e.g., **ICMJE, MIBBI, ARRIVE, PRISMA**) have been followed or provided.	Yes	Methods
For **tumor marker prognostic studies,** we recommend that you follow the **REMARK** reporting guidelines (see link list at top right). See author guidelines, under 'Reporting Guidelines'. Please confirm you have followed these guidelines.	Not Applicable	
For **phase II and III randomized controlled trials**, please refer to the **CONSORT** flow diagram (see link list at top right) and submit the CONSORT checklist (see link list at top right) with your submission. See author guidelines, under 'Reporting Guidelines'. Please confirm you have submitted this list.	Not Applicable	

Data Availability

Data availability	Information included in the manuscript?	In which section is the information available? (Reagents and Tools Table, Materials and Methods, Figures, Data Availability Section)
Have **primary datasets** been deposited according to the journal's guidelines (see 'Data Deposition' section) and the respective accession numbers provided in the Data Availability Section?	Not Applicable	
Were **human clinical and genomic datasets** deposited in a public access-controlled repository in accordance to ethical obligations to the patients and to the applicable consent agreement?	Not Applicable	
Are **computational models** that are central and integral to a study available without restrictions in a machine-readable form? Were the relevant accession numbers or links provided?	Not Applicable	
If publicly available data were reused, provide the respective **data citations in the reference list**.	Not Applicable	
